# Glycosphingolipids are linked to elevated neurotransmission and neurodegeneration in a *Drosophila* model of Niemann Pick type C

**DOI:** 10.1242/dmm.050206

**Published:** 2023-10-12

**Authors:** Anna E. Eberwein, Swarat S. Kulkarni, Emma Rushton, Kendal Broadie

**Affiliations:** ^1^ Department of Biological Sciences, Vanderbilt University and Medical Center, Nashville, TN 37235, USA; ^2^ Department of Cell and Developmental Biology, Vanderbilt University and Medical Center, Nashville, TN 37235, USA; ^3^ Vanderbilt Brain Institute, Vanderbilt University and Medical Center, Nashville, TN 37235, USA; ^4^ Kennedy Center for Research on Human Development, Vanderbilt University and Medical Center, Nashville, TN 37235, USA

**Keywords:** Lipid storage disease, Neurotransmission, Excitotoxicity, Mannosyl glucosylceramide, Glucosylceramide

## Abstract

The lipid storage disease Niemann Pick type C (NPC) causes neurodegeneration owing primarily to loss of NPC1. Here, we employed a *Drosophila* model to test links between glycosphingolipids, neurotransmission and neurodegeneration. We found that *Npc1a* nulls had elevated neurotransmission at the glutamatergic neuromuscular junction (NMJ), which was phenocopied in *brainiac* (*brn*) mutants, impairing mannosyl glucosylceramide (MacCer) glycosylation. *Npc1a; brn* double mutants had the same elevated synaptic transmission, suggesting that *Npc1a* and *brn* function within the same pathway. Glucosylceramide (GlcCer) synthase inhibition with miglustat prevented elevated neurotransmission in *Npc1a* and *brn* mutants, further suggesting epistasis. Synaptic MacCer did not accumulate in the NPC model, but GlcCer levels were increased, suggesting that GlcCer is responsible for the elevated synaptic transmission. Null *Npc1a* mutants had heightened neurodegeneration, but no significant motor neuron or glial cell death, indicating that dying cells are interneurons and that elevated neurotransmission precedes neurodegeneration. Glycosphingolipid synthesis mutants also had greatly heightened neurodegeneration, with similar neurodegeneration in *Npc1a; brn* double mutants, again suggesting that *Npc1a* and *brn* function in the same pathway. These findings indicate causal links between glycosphingolipid-dependent neurotransmission and neurodegeneration in this NPC disease model.

## Teaser

**Summary:** Genetic analyses in a *Drosophila* disease model show that loss of Npc1 and glycosphingolipid pathway synthesis elevate glutamatergic neurotransmission within a common functional pathway, and this pathway links to subsequent neurodegeneration.

## INTRODUCTION

Niemann Pick diseases are lysosomal lipid storage disorders causing early-onset neurodegeneration and childhood death (Niemann, 1914; Pick, 1933; Liu et al., 2020). Niemann Pick type C (NPC) is characterized by lysosomal accumulation of cholesterol and glycosphingolipids (GSLs) (Brady et al., 1966; Newton et al., 2020; Wanikawa et al., 2019). Ninety-five percent of NPC cases are caused by variants in the *NPC1* gene (Carstea et al., 1997), encoding an endolysosomal 13-pass transmembrane protein containing a sterol-sensing domain (Iannou, 2000; Long et al., 2020; Millard et al., 2005). Although cholesterol is widely considered to be the primary pathogenic lipid, efforts to disrupt low-density lipoprotein uptake do not impact NPC progressive neurodegeneration (Erickson et al., 2000; Newton et al., 2018). In contrast, miglustat inhibition of glucosylceramide (GlcCer) synthase, the first committed step in the GSL synthesis pathway, clinically stabilizes NPC patient symptoms (Patterson et al., 2007, 2020; Platt et al., 1994). Moreover, miglustat reverses hippocampal glutamatergic neurotransmission hyperexcitability in NPC disease model mice, and miglustat blocks Purkinje neuron death in NPC disease model cats (D'Arcangelo et al., 2016; Stein et al., 2012). These exciting advances suggest that the GSL synthesis pathway could be key to understanding the NPC condition, and that GSL impairments may provide the causal mechanistic link between neurotransmission and neurodegeneration defects.

As in NPC patients and other disease models, the *Drosophila* NPC model shows neuronal cholesterol accumulation, impaired synaptic transmission, movement defects, neurodegeneration and shortened lifespan (Huang et al., 2005; Phillips et al., 2008). *Drosophila* has two Npc1 genes, with *Npc1a* widely expressed throughout the body but *Npc1b* only expressed within the midgut (Fluegel et al., 2006; Huang et al., 2005). In the *Drosophila* system, the neuromuscular junction (NMJ) is a well-characterized model glutamatergic synapse (Broadie and Bate, 1995; Chou et al., 2020). Mouse hippocampal glutamatergic synapses manifest dysfunction in the absence of *Npc1* (Cariati et al., 2021; D'Arcangelo et al., 2011, 2016; Mitroi et al., 2019). Previous studies show that NPC model mice exhibit an increase in hippocampal synaptic transmission and defects in long-term potentiation (LTP) (D'Arcangelo et al., 2011, 2016; Mitroi et al., 2019), with links to cholesterol accumulation but little study of possible GSL contributions. The *Drosophila* NMJ provides a powerful genetic system for understanding the lipid storage mechanism of synaptic dysfunction through genetic manipulation of the GSL biosynthetic pathway. Critically, GSLs are established to regulate *Drosophila* NMJ structure/function (Huang et al., 2018), and GSLs are known to activate apoptosis through inducing autophagy, endoplasmic reticulum stress, lysosomal membrane permeabilization and the release of pro-apoptotic mitochondrial proteins (Garcia-Ruiz et al., 2015; Park and Park, 2020; Rippo et al., 2000; Wheeler et al., 2019). These studies suggest a possible link between GSL and neurotransmission and neurodegeneration defects.

*Drosophila* mutants in serine palmitoyl transferase (*lace* gene), the rate-limiting step in sphingolipid synthesis, exhibit elevated NMJ neurotransmission from fewer NMJ synaptic boutons and also display increased apoptosis (Kraut, 2011; West et al., 2018). Likewise, mannosyl glucosylceramide (MacCer), a GSL synthesis pathway intermediate similar to mammalian lactosylceramide (LacCer), regulates NMJ structure and *trans*-synaptic signaling (Huang et al., 2018; Wandall et al., 2005). Other GSLs are also implicated in conserved pathogenic phenotypes in several models of neurodegenerative diseases. GlcCer accumulation is thought to be the underlying cause of spatial ataxia in Gaucher's disease, caused by loss of glucocerebrosidase 1 (GBA1), the endolysosomal enzyme that de-glycosylates GlcCer (Malekkou et al., 2018). Heterozygous variation in GBA1 is a leading genetic risk factor for Parkinson's disease, characterized by both motor ataxia and progressive neurodegeneration, similar to the NPC state (Stoker et al., 2020). Accumulated GlcCer storage within the lysosome is also thought to lead to an increase in the lumenal pH (Sillence, 2013), and lysosomal de-acidification is well known to result in perforations in the organelle membrane, which cause mitochondrial oxidative stress and activate an intrinsic apoptotic pathway (Vest et al., 2022; Wheeler and Sillence, 2020). We therefore hypothesized that similar GSL trafficking accumulation defects cause elevated glutamatergic neurotransmission and neurodegeneration in NPC.

To test this hypothesis, we used mutants in *brainiac* (*brn*), encoding the beta-1,3-N-acetylglucosaminyltransferase that glycosylates MacCer (Wandall et al., 2005), as well as *egghead* (*egh*), encoding the beta-4-mannosyltransferase that glycosylates GlcCer to MacCer (Wandall et al., 2005). Using two-electrode voltage-clamp (TEVC) recording at the NMJ, we found that *Npc1a* and *brn* mutants have elevated glutamatergic transmission, but observed no change in *egh* mutants. Neuronal RNA interference (RNAi) knockdown of *Npc1a* and *brn* demonstrated a presynaptic requirement. In *Npc1a; brn* double mutants, we found no further increase in neurotransmission, indicating that the two genes operate within the same pathway. We further found that *Npc1a; egh* double mutants had elevated transmission comparable to that in *Npc1a* nulls alone, suggesting that MacCer is not responsible for synaptic dysfunction. Consistently, the GlcCer synthase inhibitor miglustat blocked elevated neurotransmission in *Npc1a* and *brn* mutants. MacCer labeling showed no synaptic accumulation in *Npc1a* nulls, but maintained elevation in *Npc1a; brn* double mutants. Moreover, *Npc1a* mutants had increased GlcCer levels, suggesting that GlcCer is likely to be pathogenic. Assaying neurodegeneration with terminal deoxynucleotidyl transferase biotin-dUTP nick end labeling (TUNEL) labeling (Vita et al., 2021) revealed that *Npc1a* nulls had heightened neuronal death, with synaptic dysfunction preceding neurodegeneration in motor neurons. Consistently, neuronal death was strongly elevated in *egh* and *brn* mutants, and similarly increased in *Npc1a; brn* double mutants. These findings causally link GSL accumulation to elevated neurotransmission and neurodegeneration in the *Drosophila* NPC disease model.

## RESULTS

### *Npc1a* null mutants show elevated glutamatergic neurotransmission at the NMJ

**Fig. 1.**
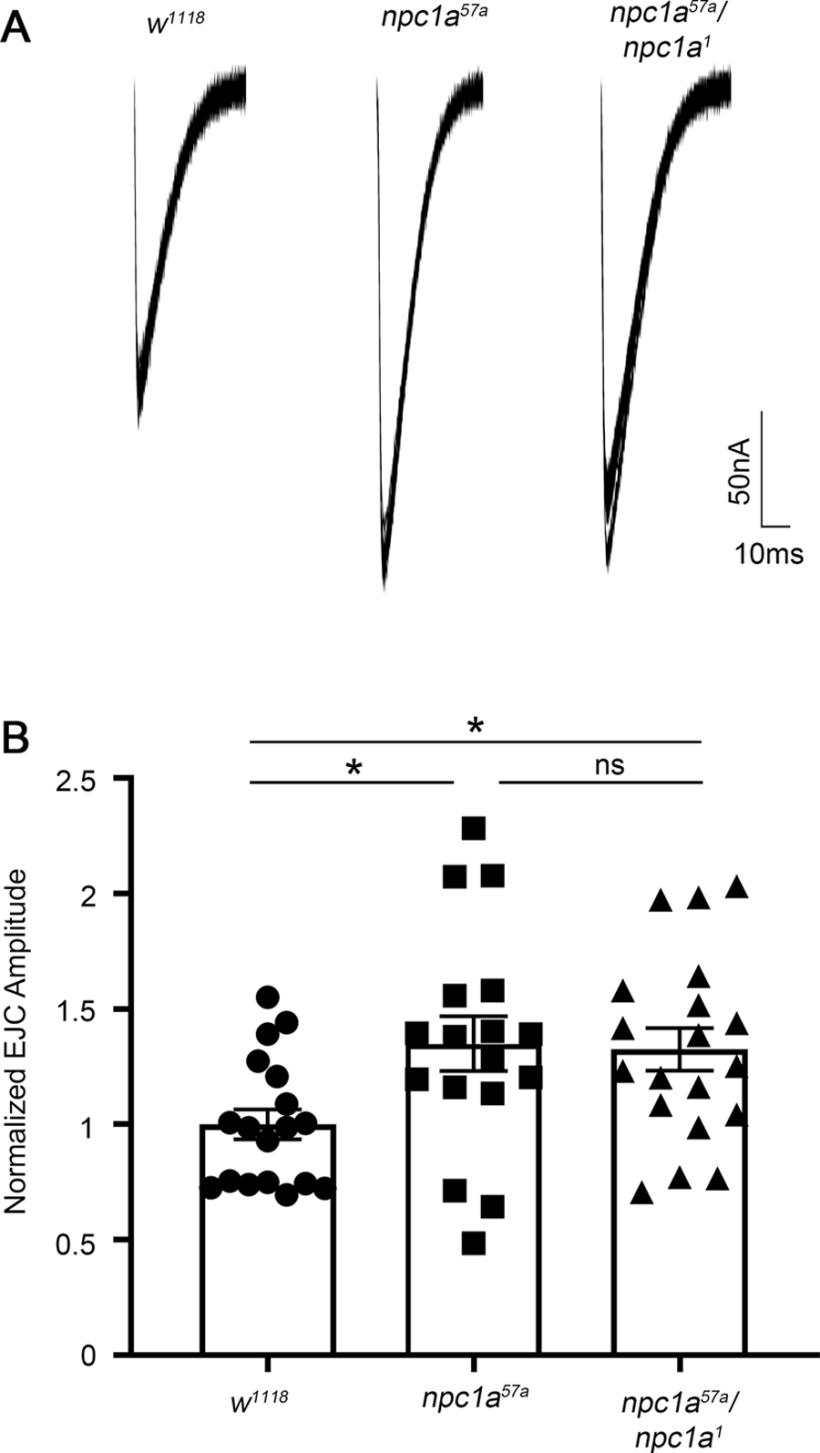
***Npc1a* null mutants display elevated glutamatergic neurotransmission.** (A) Representative excitatory junction current (EJC) traces showing ten superimposed two-electrode voltage-clamp (TEVC) recordings from the *Drosophila* wandering third-instar neuromuscular junction (NMJ) of the genetic background control (*w^1118^*, left), the homozygous *Npc1a* null mutant (*Npc1a^57a^*, middle) and the *trans*-heterozygous null mutant (*Npc1a^57a^/Npc1a^1^*, right). (B) Quantified EJC amplitudes normalized to the *w^1118^* control (*n*=18), compared to *Npc1a^57a^/Npc1a^57a^* (*n*=17) and *Npc1a^57a^/Npc1a^1^* (*n*=19) mutants. One-way ANOVA and Tukey's multiple comparison tests show that both mutant conditions are significantly elevated compared to control (*P=0.031 and *P=0.041, respectively), with no significant (ns) difference between the two mutants. All graphs show scatter plots of all individual data points with the mean±s.e.m.

To test synaptic transmission strength in the *Drosophila* model of NPC disease, TEVC electrophysiology was used to assay glutamatergic signaling at the well-characterized NMJ (Bhimreddy et al., 2021). Null *Npc1a* mutants were fed 7-dehydrocholesterol (0.14 mg/g^−1^) as an ecdysone precursor to circumvent the early larval lethality resulting from the lack of steroid hormone signaling (Huang et al., 2005; Phillips et al., 2008). Alternatively, the *2-286* Gal4 driver was used to express UAS-*Npc1a* in the endocrine ring gland, the site of ecdysone steroid synthesis (Phillips et al., 2008). The approaches produced identical results. Homozygous *Npc1a^57a^* and *trans*-heterozygous *Npc1a^57a^/Npc1a^1^* nulls (Fluegel et al., 2006; Huang et al., 2005) were compared to genetic background control (*w^1118^*). The *Npc1a^57a^; 2-286* Gal4>UAS*-Npc1a::YFP* line was compared to the *2-286* Gal4 driver alone control (*2-286* Gal4/*+*). Electrical stimulation of the wandering third-instar motor nerve with a glass suction electrode in 1.5 mM [Ca^2+^] was used to drive NMJ glutamatergic transmission onto ventral longitudinal muscle 6 (abdominal segments 3/4), voltage clamped at −60 mV. To assay synaptic transmission strength, ten consecutive excitatory junction current (EJC) traces were collected (0.2 Hz) from each NMJ and then averaged for each single data point (Bhimreddy et al., 2021; Kopke et al., 2017). Representative synaptic electrophysiology recordings and quantified results are shown in Fig. 1.

Repeated nerve stimulations resulted in strong, consistent EJC responses with high-fidelity amplitudes (Fig. 1A). The *Drosophila* NPC disease model of *Npc1a* null mutants showed strikingly increased glutamatergic currents compared to matched genetic controls. Synaptic transmission was similarly elevated in both *Npc1a^57a^* and *Npc1a^57a^/Npc1a^1^* nulls compared with the *w^1118^* genetic background controls (Fig. 1A). The normalized EJC amplitude for the control is 1.00±0.07 (mean±s.e.m.; *n*=18; Fig. 1B) compared to the homozygous *Npc1a^57a^* mutant elevated response of 1.35±0.12 (*n*=17; Fig. 1B). Likewise, *trans*-heterozygous *Npc1a^57a^/Npc1a^1^* mutants showed the same elevation, with an average EJC amplitude of 1.32±0.09 (*n*=19; Fig. 1A,B). EJC amplitudes of homozygous *Npc1a^57a^* and heterozygous *Npc1a^57a^/Npc1a^1^* were not significantly different (*P*=0.980), but both were significantly increased from that of the control (*P*=0.031, *P*=0.041, respectively; Fig. 1B). Ring gland-rescued *Npc1a* nulls (*Npc1a^57a^; 2-286* Gal4>UAS*-Npc1a::YFP*) closely phenocopied the 7-dehydrocholesterol-fed EJC amplitude elevation (Fig. S1A), with a normalized EJC amplitude of 1.26±0.13 (*n*=18) compared to that of the *2-286* Gal4/*+* control of 1.00±0.059 (*n*=13, *P*=0.046; Fig. S1B), indicating that cholesterol supplementation does not impact the increased neurotransmission. Taken together, these findings demonstrate that the *Drosophila* NPC disease model shows elevated glutamatergic synaptic transmission independent of cholesterol feeding. We therefore next tested whether this functional neurotransmission defect could be explained by a GSL mechanism.

### GSL synthesis mutants phenocopy the elevated synaptic transmission observed in *Npc1a* mutants

The NPC lipid storage disease state involves GSL mistrafficking and accumulation (te Vruchte et al., 2004; Wheeler et al., 2019; Zervas et al., 2001a,b), and GSL pathway mutants provide a way of testing the neurotransmission requirements (Huang et al., 2018; Newton et al., 2020). There are well-established links between GSL accumulation and elevated glutamatergic signaling in NPC models (D'Archangelo et al., 2016). We therefore hypothesized that GSL pathway mutants would phenocopy our NPC disease model synaptic defects. Previous studies have shown that *brn* mutants (*brn^fs.107^*) of beta-1,3-N-acetylglucosaminyltransferase that glycosylates MacCer (Wandall et al., 2005) accumulate MacCer at the *Drosophila* NMJ, which is linked to NMJ overgrowth (Huang et al., 2018). To test parallels in the NPC model, NMJs were co-labeled with anti-horseradish peroxidase (HRP), which recognizes fucosylated N-glycans on the presynaptic neuronal membrane (Jan and Jan, 1982; Kopke et al., 2017), and anti-Discs Large (DLG; also known as Dlg1), which labels the postsynaptic scaffold in the subsynaptic reticulum (Lahey et al., 1994; Kopke et al., 2017). Like *brn* mutants, *Npc1a* mutants showed NMJ overgrowth (Fig. S2A). Compared to controls, the mutants had significantly increased NMJ area (*P*<0.0001; Fig. S2B) and synaptic bouton number (*P*<0.0001; Fig. S2C). However, there was no change in satellite bouton number (Fig. S2D) and only a small increase in synaptic branch number (*P*=0.0159; Fig. S2E). There was no change in the presynaptic marker Bruchpilot (Brp) marking synapses (Kittel et al., 2006), suggesting that the NMJ overgrowth is not coupled to neurotransmission function.

**Fig. 2.**
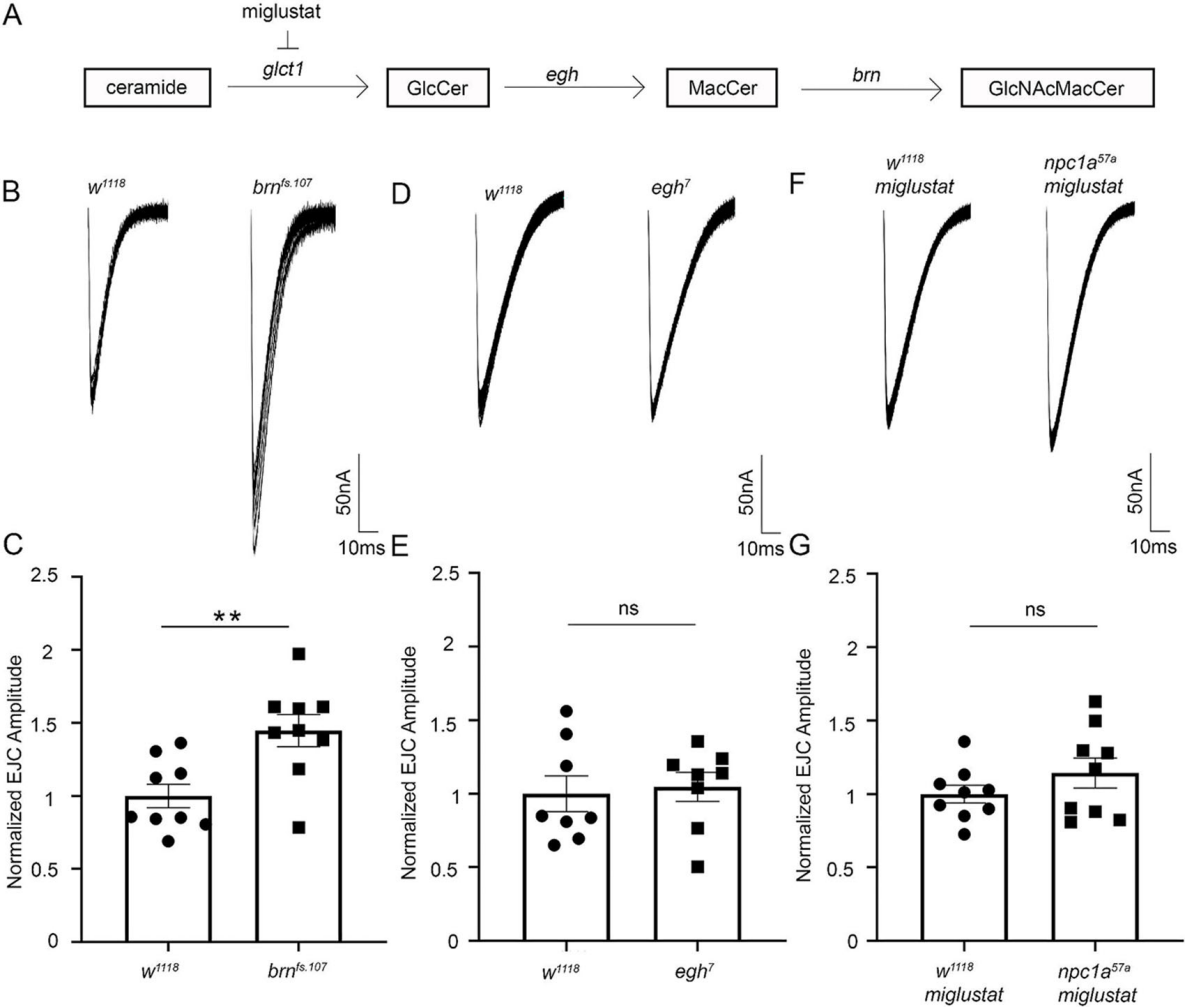
**Glycosphingolipid manipulations differentially impact neurotransmission.** (A) Schematic of the glycosphingolipid biosynthetic pathway. (B) Representative EJC traces (ten superimposed recordings) from the genetic control (*w^1118^*, left) and *brn* mutant (*brn^fs.107^*, right). (C) Normalized EJC amplitudes for control (*n*=9) and *brn^fs.107^* (*n*=9) show a significant increase based on unpaired two-tailed Student’s *t*-test (**P=0.0045), phenocopying the *Npc1a* null. (D) EJC traces for *w^1118^* (left) and *egh^7^* (right). (E) Quantified EJC amplitudes for control (*n*=8) and *egh^7^* (*n*=8) show no significant (ns) difference (*P*=0.771) based on an unpaired two-tailed Student's *t*-test. (F) EJC traces of *w^1118^* (left) and *Npc1a^57a^* homozygous null mutants (right) fed from hatching with miglustat (0.1 ng/ml) to inhibit glucosylceramide synthase. (G) Quantified EJC amplitudes normalized to *w^1118^* on miglustat (*n*=9) compared to *Npc1a^57a^* on miglustat (*n*=9) show no significant (ns) difference (*P*=0.2436) based on an unpaired two-tailed Student's *t*-test. GlcCer, glucosylceramide; MacCer, mannosyl glucosylceramide; GlcNAcMacCer, N-acetylglucosamine mannosyl glucosylceramide.

To test the connection between GSL pathway and NPC model neurotransmission, we took TEVC recordings of *brn* mutants as well as *egh* mutants of the beta4-mannosyltransferase enzyme, which glycosylates GlcCer to MacCer (Fig. 2A). Previous studies have shown that *egh^7^* mutants accumulate GlcCer (Wandall et al., 2005). In parallel, we used the GlcCer synthase inhibitor miglustat (D'Archangelo et al., 2016; Patterson et al., 2020) to block ceramide glycosylation (Fig. 2A). Compared to control (*w^1118^*) normalized EJC amplitude (1.00±0.08, *n*=9), *brn^fs.107^* mutants had increased synaptic transmission (1.45±0.11, *n*=9), which was significantly elevated (*P*=0.0045; Fig. 2B,C) and phenocopied that of *Npc1a* mutants. In contrast, *egh^7^* mutants showed no increase in EJC amplitude compared to that of controls (Fig. 2D,E). We therefore next tested whether the GlcCer synthase inhibitor miglustat affects the *Npc1a* phenotype. Feeding mutant animals miglustat prevented the elevated synaptic transmission (Fig. 2F). Compared to miglustat-fed control EJC amplitudes of 1.00±0.06 (*n*=9), miglustat-fed *Npc1a* nulls had an EJC amplitude of 1.14±0.10 (*n*=9), which was not significantly different from that of controls (*P*=0.247; Fig. 2G). Likewise, the miglustat-fed *brn^fs.107^* mutant EJC amplitude of 1.07±0.09 (*n*=10) was not significantly different from that of controls (*P*=0.3494; Fig. S3A,B). Importantly, these results establish that GSL accumulation causes a strong increase in NMJ neurotransmission, and that the GSL synthesis pathway is causative of the increase in NPC glutamatergic synaptic function. We next tested whether *Npc1a* and *brn* function within the same cell compartment to increase synaptic transmission.

**Fig. 3.**
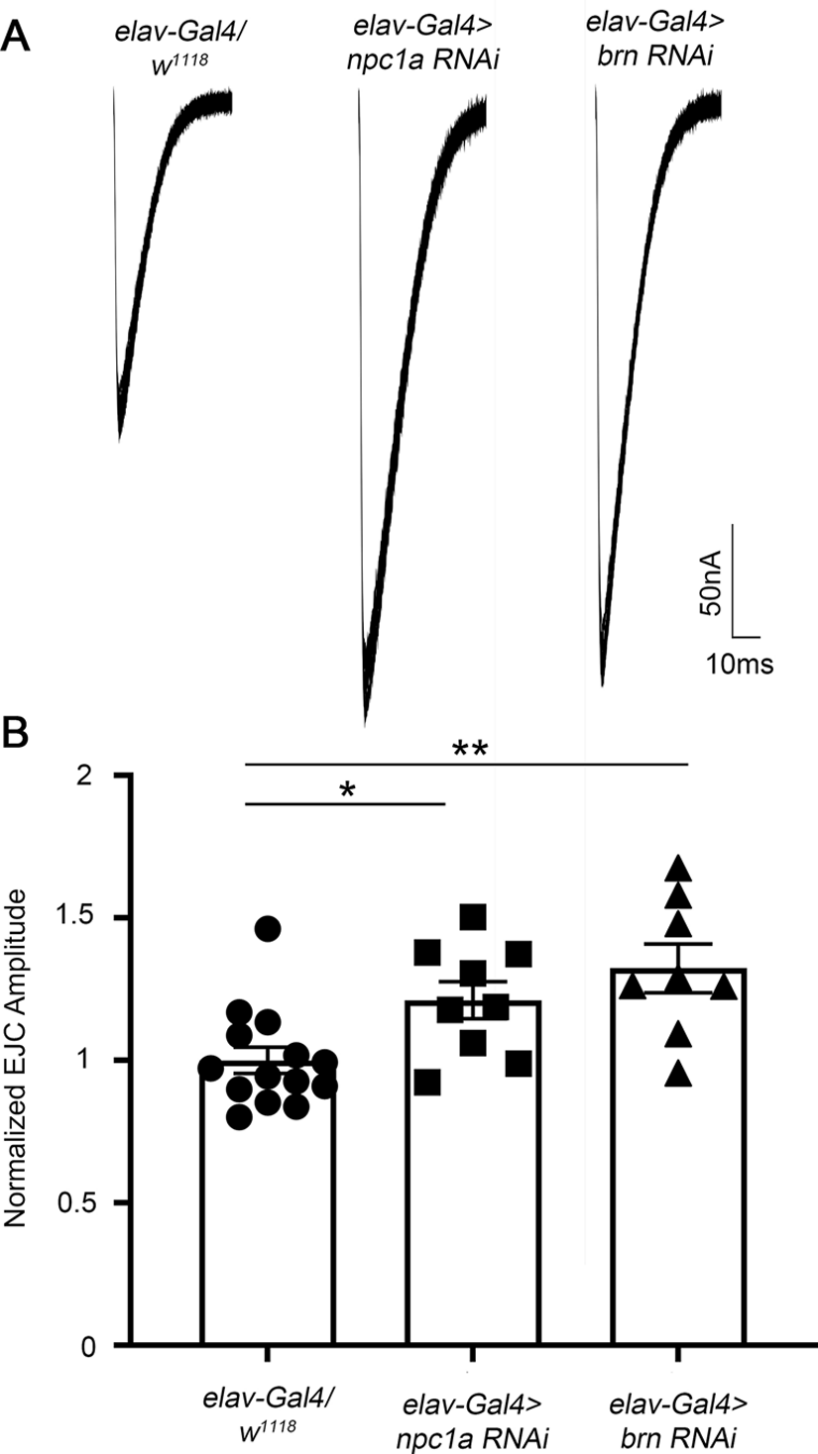
**Neuron RNA interference (RNAi) knockdown of *Npc1a* and *brn* increases neurotransmission.** (A) Representative EJC traces (ten superimposed recordings) from the neuronal Gal4 driver control alone (*elav*-Gal4/*w^1118^*, left), and driving *Npc1a* RNAi (*elav*-Gal4*>Npc1a* RNAi, middle) and *brn* RNAi (*elav*-Gal4*>brn* RNAi, right). (B) EJC amplitudes normalized to the driver control show a significant increase for neuronal *elav*-Gal4*>Npc1a* RNAi (*n*=9, *P=0.036) and more significant increase for neuronal *elav*-Gal4*>brn* RNAi (*n*=8, **P=0.0043) compared to the matched driver control (*n*=14) based on a Kruskal–Wallis test with multiple comparisons.

Previous studies of NPC model synapses revealed presynaptic and postsynaptic defects that can impact neurotransmission strength (Hawes et al., 2010; Mitroi et al., 2019). We therefore next tested the cell type requirements for the increase in synaptic transmission in the *Npc1a* and *brn* mutants. We used a targeted Gal4 driver specific to neurons (*elav-*Gal4) to drive both *Npc1a* and *brn* RNAi knockdown, together with TEVC recording at the NMJ to measure changes in glutamatergic neurotransmission. Compared to the transgenic driver alone control (*elav*-Gal4*/w^1118^*), targeted neuronal knockdown of both *Npc1a* and *brn* increased synaptic function (Fig. 3A). Neuron-specific *Npc1a* RNAi elevated the EJC amplitude to 1.211±0.07 (*n*=9), normalized to the control 1.00±0.05 (*n*=14), which was a significant increase (*P*=0.036; Fig. 3B). Likewise, *elav-*Gal4*-*driven *brn* RNAi elevated synaptic transmission to 1.323±0.09 (*n*=8), a significant increase compared to that of control (*P*=0.0043), phenocopying the NPC model (Fig. 3B). These results establish that neuronal knockdown of either *Npc1a* or *brn* is sufficient to induce the increase in neurotransmission, indicating that changes in the presynaptic neuron are responsible for the strengthened synaptic transmission. The finding that both *Npc1a* and *brn* similarly impact presynaptic neurotransmission supports the hypothesis that *Npc1a* and *brn* act in the same pathway regulating synaptic function. We therefore next tested for genetic interactions between the GSL synthesis pathway and the NPC disease model.

### *Npc1a* and *brn* function in the same pathway to increase neurotransmission

**Fig. 4.**
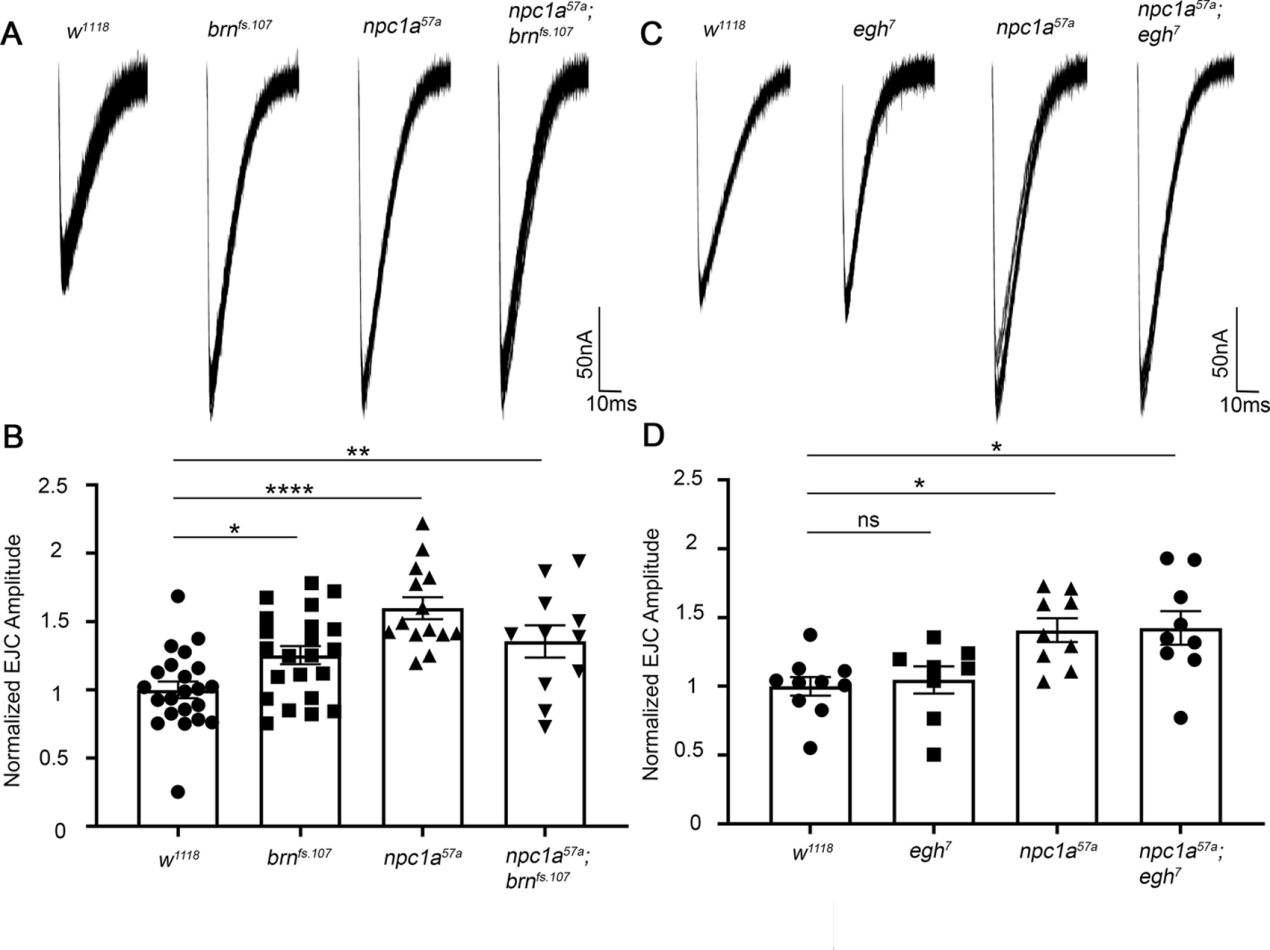
***Npc1a* and *brn* function in same pathway to increase neurotransmission.** (A) Representative EJCs for genetic control (*w^1118^*), *brn* (*brn^fs.107^*), *Npc1a* (*Npc1a^57a^*) and the double mutant (*Npc1a^57a^; brn^fs.107^*). (B) Quantified EJC amplitude normalized to control (*n*=22), for *brn^fs.107^* (*n*=22), *Npc1a^57a^* (*n*=14) and double mutants (*n*=11). All are significantly increased based on one-way ANOVA and Dunnett's multiple comparisons tests: *brn^fs.107^* (*P=0.027), *Npc1a^57^* (****P<0.0001) and *Npc1a^57a^; brn^fs.107^* (**P=0.0098). (C) EJCs for control (*w^1118^*), *egh* mutant (*egh^7^*), *Npc1a^57^* mutant and double mutant (*Npc1a^57^; egh^7^*). (D) The quantified EJC amplitudes normalized to control (*n*=10), for *egh^7^* (*n=8*), *Npc1a^57a^* (*n*=9) and *Npc1a^57^; egh^7^* (*n*=9). Both *Npc1a^57a^* and *Npc1a^57^; egh^7^* are significantly increased based on one-way ANOVA with Tukey's multiple comparison tests (*P=0.0188, *P=0.0140, respectively), with no significant (ns) difference between them.

Double mutant genetic combinations can be used to test functional interactions in a common versus a parallel mechanism (Haber et al., 2013; Slade and Staveley, 2015). For this test, we first created *Npc1a; brn* double mutants to test synaptic transmission phenotypes compared to each single mutant and the *w^1118^* genetic background control. We hypothesized that if the *Npc1a; brn* double mutants were to display the same elevated neurotransmission as each single mutant alone, then t"he two gene products must act in a common pathway regulating synaptic signaling strength. To further test GSLs upstream of *brn* and downstream of miglustat inhibition (MacCer and GlcCer) (Huang et al., 2018; Wandall et al., 2005), we next created *Npc1a; egh* double mutants to determine whether *egh* loss of function modulates the synaptic transmission elevation in our NPC disease model. We hypothesized that if the *Npc1a; egh* double mutants exhibit a restored synaptic transmission amplitude, similar to the above miglustat result (Fig. 2F,G), then GSLs downstream of GlcCer would be causative of the increased synaptic transmission in the *Npc1a* null mutants. In all combinations, we used the same rearing methods as above, including feeding 7-dehydrocholesterol to homozygous *Npc1a^57a^* (Phillips et al., 2008), *brn^fs.107^* and *egh^7^* mutants (Wandall et al., 2005), and the double mutants. As above, we performed TEVC electrophysiological recordings at the third-instar NMJs. Representative traces and quantified data are shown in Fig. 4.

We first tested *Npc1a^57a^; brn^fs.107^* double mutants, to find the same highly elevated synaptic transmission as in each single homozygous *Npc1a^57a^* and *brn^fs.107^* mutants alone (Fig. 4A). Normalized to the *w^1118^* genetic background control, the *brn^fs.107^* mutants showed a mean EJC amplitude of 1.25±0.07 (*n*=22), which was significantly increased compared to that of matched controls (*P*=0.027; Fig. 4A,B). Likewise, *Npc1a^57a^* mutants had a normalized EJC amplitude of 1.60±0.08 (*n*=14), which was also significantly elevated compared to that of *w^1118^* controls (*P*<0.0001; Fig. 4A,B). Importantly, *Npc1a^57a^; brn^fs.107^* double mutants showed an intermediate mean EJC amplitude of 1.36±0.12 (*n*=11), with a similar significant increase compared to that of the controls (*P*=0.0098; Fig. 4A,B). Moreover, the mean EJC amplitude of the *Npc1a^57a^; brn^fs.107^* double mutants was not significantly increased from that of the *Npc1a^57a^* (*P*=0.235) or the *brn^fs.107^* (*P*=0.824) single mutant. These results indicate that *brn* and *Npc1a* function in the same pathway to increase synaptic transmission, as opposed to different pathways that would cause an additive increase in the double mutant EJC amplitude (Fig. 4A,B). Miglustat feeding, which inhibits GlcCer synthase, prevented the increase in synaptic transmission in our NPC disease model (Fig. 2E,F), and the *Npc1a^57a^; brn^fs.107^* double mutants, which have blocked GSL synthesis past MacCer, showed no change in the increased synaptic transmission (Fig. 4A,B); these results together suggest that MacCer or GlcCer may be causative of *Npc1a*-increased synaptic transmission, and that *egh^7^* mutants that inhibit GlcCer to MacCer synthesis may be able to rectify the *Npc1a* mutant increase in neurotransmission.

We therefore next tested *Npc1a^57a^; egh^7^* double mutants, to find the same highly elevated neurotransmission as in the single homozygous *Npc1a^57a^* mutant alone (Fig. 4C). Both *Npc1a^57a^* single mutants and *Npc1a^57a^; egh^7^* double mutants showed indistinguishably increased synaptic transmission compared with the *w^1118^* genetic background control. Normalized to control (1.00±0.07; *n*=10), the EJC amplitude of the *Npc1a^57a^* single mutant was 1.408±0.09 (*n*=9) and that of the *Npc1a^57a^; egh^7^* double mutant was 1.42±0.12 (*n*=9), and both were significantly increased from that of *w^1118^* (*P*=0.0188 and *P*=0.0140, respectively; Fig. 4D). Importantly, there was no significant difference between the *Npc1a^57a^* single mutant and *Npc1a^57a^; egh^7^* double mutant (*P*=0.9994; Fig. 4D). Because the *egh^7^* mutants alone exhibited no change in synaptic transmission strength (1.046±0.10; *n*=8; *P*=0.9859; Fig. 4D), and the double mutants showed no change from the single *Npc1a^57a^* mutant (Fig. 4C,D), we can make no firm conclusion regarding the causative influence of GlcCer on the neurotransmission elevation in our NPC model. Therefore, GlcCer and/or MacCer may be the causative, pathogenic GSL underlying the synaptic transmission phenotype, but electrophysiological recording alone has so far been unable to distinguish these possibilities. To address this critical question, we next used a characterized anti-MacCer antibody (Wandall et al., 2005) to visualize and quantify synaptic MacCer levels at the NMJ to determine whether MacCer levels correlate with the synaptic transmission levels in our panel of mutants.

### Synaptic MacCer is normal in *Npc1a* mutants but elevated in *Npc1a; brn* double mutants

**Fig. 5.**
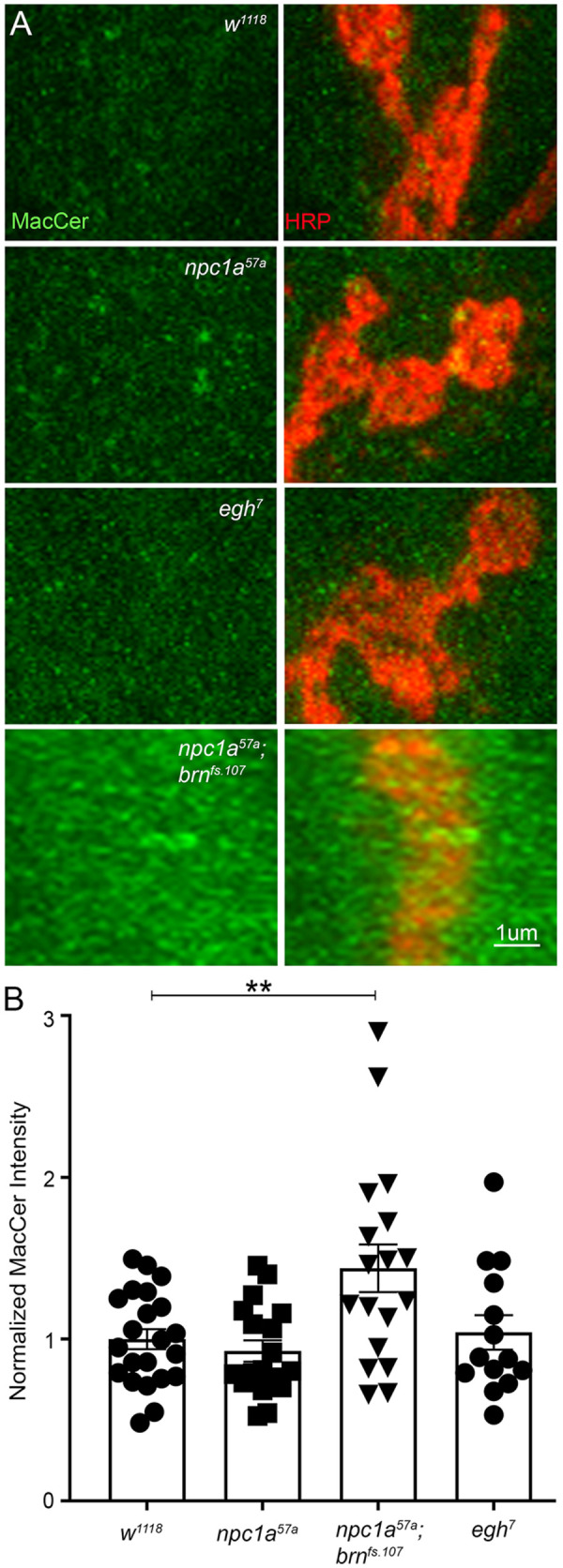
***Npc1a* displays normal synaptic MacCer, elevated in *Npc1a; brn* mutants.** (A) Representative confocal images of anti-MacCer (green) co-labeled with the synaptic membrane marker anti-horseradish peroxidase (HRP; red) at the wandering third-instar NMJ in the genetic background control (*w^1118^*, top row), *Npc1a^57^* mutant (second row), *egh^7^* mutant (third row) and *Npc1a^57a^; brn^fs.107^* double mutant (bottom row). MacCer labeling alone (green) is shown in the left column, with the HRP synaptic marker (red) in the right column. (B) Quantification of MacCer fluorescent intensity normalized to the *w^1118^* control (*n*=22), compared to *Npc1a^57^* (*n*=18), *egh^7^* (*n*=14) and *Npc1a^57a^; brn^fs.107^* (*n*=18), shows only the double mutant to be significantly elevated compared to the control based on one-way ANOVA with Tukey's multiple comparison tests (**P=0.0025). There are no significant (ns) changes in the single mutants compared to control.

Previous studies have reported the accumulation of LacCer, the mammalian homolog of *Drosophila* MacCer, in mouse NPC model brains as well as in NPC patient fibroblasts (Colaco et al., 2019; te Vruchte et al., 2004). In light of the importance of MacCer at the *Drosophila* NMJ synaptic terminal (Huang et al., 2018), we hypothesized that MacCer accumulation would be responsible for the elevated synaptic transmission in our *Drosophila* NPC model. In order to test for MacCer accumulation defects at the NMJ, we used a characterized anti-MacCer antibody for confocal imaging (Wandall et al., 2005). We tested synaptic MacCer levels in the genetic background controls (*w^1118^*), *Npc1a* nulls (*Npc1a^57a^*), GSL pathway mutants (*brn^fs.107^*, *egh^7^*) and *Npc1a^57a^; brn^fs.107^* double mutants. Previous work has shown an increase in MacCer in *brn^fs.107^*/*brn^1.6P6^* mutants and decrease in MacCer in *egh^62d18^* mutants at the *Drosophila* NMJ (Huang et al., 2018). We co-labeled NMJs with anti-MacCer and anti-HRP antibody, which labels the presynaptic neuronal membrane (Jan and Jan, 1982; Kopke et al., 2017). We then measured anti-MacCer fluorescent intensity at HRP-positive NMJ synaptic boutons on muscle 4 (abdominal segments 3,4) (Kopke et al., 2017; Roos et al., 2000). Measurements were made in and around the large type 1b synaptic boutons (Knodel et al., 2014), including presynaptic neuronal and postsynaptic muscle membrane compartments. Representative images and quantifications are shown in Fig. 5.

In *w^1118^* genetic control NMJs, MacCer localized in punctate accumulations in both presynaptic and postsynaptic regions (Fig. 5A, top row). Similar low-level MacCer labeling was present in both *Npc1a^57a^* and *egh^7^* mutants, which were indistinguishable from controls (Fig. 5A, middle row). Normalized to *w^1118^* controls (1.00±0.06, *n*=22), *Npc1a^57a^* nulls showed a mean fluorescent intensity of 0.93±0.07 (*n*=18), which is not different from that of controls (*P*=0.944; Fig. 5B). Given that MacCer levels appear identical in *Npc1a^57a^* mutants and matched controls, synaptic MacCer is not a good candidate to explain the elevated synaptic transmission in our NPC model. Moreover, we found no difference in MacCer levels at *egh^7^* mutant NMJs (1.04±0.11; *n*=14; *P*=0.9915; Fig. 5B), which differs from the previously reported decrease in *egh^62d18^* and *egh^7^* mutants in whole-larva thin-layer chromatography analyses (Huang et al., 2018; Wandall et al., 2005). We note that the *egh^62d18^* allele is a frameshift that deletes most of the *egh* coding sequence, compared to the *egh^7^* single point mutation (Wandall et al., 2005), so *egh^7^* may not be strong enough to show the same phenotype. The *egh^62d18^* mutant appears to be lost based on our extensive efforts to locate this line. Low levels of synaptic MacCer in genetic background controls (Fig. 5A) may make it difficult to quantify the MacCer loss present in *egh^7^* whole-larva analyses (Wandall et al., 2005). We expect that *egh^7^* mutants do exhibit decreased MacCer levels, consistent with the reported loss of enzymatic function.

*brn^fs.107^* mutants have previously been shown to exhibit strongly elevated MacCer at the *Drosophila* NMJ (Huang et al., 2018). Similarly, *Npc1a^57a^; brn^fs.107^* double mutants showed a very striking increase in synaptic MacCer accumulation compared to genetic controls (Fig. 5A, bottom row). The synaptic MacCer elevation in *brn^fs.107^* mutants and *Npc1a^57a^; brn^fs.107^* double mutants was apparent in larger and far more numerous punctate accumulations in both the presynaptic and postsynaptic regions, but the higher MacCer levels were not restricted to the NMJ. In both genotypes, MacCer was broadly elevated in the muscle extending outside the synaptic domain (Fig. 5A, bottom row; Fig. S4A). We confirmed that *brn^fs.107^* mutants had an increase in MacCer fluorescence intensity of 1.60±0.28 (*n*=9) normalized to the *w^1118^* genetic background control fluorescent level of 1.00±0.07 (*n*=10), a significant elevation in the mutant (*P*=0.0465; Fig. S4B). Likewise, compared to the *w^1118^* control fluorescent level of 1.00±0.06 (*n*=22), the *Npc1a^57a^; brn^fs.107^* double mutants exhibited a mean synaptic MacCer intensity of 1.44±0.15 (*n*=18), which is significantly increased compared to that of control (*P*=0.0079; Fig. 5B). Given that the *Npc1a^57a^; brn^fs.107^* double mutants showed synaptic transmission strength comparable to that of *Npc1a^57a^* mutants (Fig. 4A,B), but the double mutants displayed a striking MacCer accumulation whereas the *Npc1a^57a^* mutants did not (Fig. 5A,B), we conclude that MacCer is not a good candidate to explain the elevated synaptic transmission in our NPC model.

We therefore turned to GlcCer as the best candidate pathogenic lipid for the NPC model phenotypes. However, we lack GlcCer imaging capabilities at the *Drosophila* NMJ. As a consequence, we performed liquid chromatography–mass spectrometry (LC-MS) to assay the overall GlcCer levels (see Materials and Methods). Levels of HexCer_NS(35;3;2O) were significantly increased in the NPC model (*Npc1a^57a^* nulls), with a measured magnitude of 23,146±1320 (*n*=5 trials) compared to the *w^1118^* genetic background control with a normalized level of 14,812±1174 (*n*=5), which is a significant elevation (*P*=0.00157; Fig. S5A). Similarly, the levels of HexCer_NS(32;3;2O) were significantly increased in the *Npc1a^57a^* mutants, with a magnitude of 61,054±5541 (*n*=5) compared to *w^1118^* controls with a normalized level of 39,561±6460 (*n*=5, *P*=0.0407; Fig. S5B). Because *Drosophila* do not contain any galactosylceramide (Wandall et al., 2005), we can infer that these HexCer species must correspond to GlcCer variants. The observation that GlcCer is accumulated in the *Npc1a* null *Drosophila* NPC disease model, but MacCer is not, suggests that GlcCer is most likely to be the pathogenic lipid in this system underlying the above synaptic phenotypes. We next tested whether heightened neurotransmission excitotoxicity provides a mechanistic explanation for the subsequent neurodegeneration in the *Drosophila* NPC disease model. To test for GSL roles, we assayed neurodegeneration in the *Npc1a* and GSL pathway mutants.

### Heightened neurodegeneration in *Npc1a* and GSL pathway mutants

**Fig. 6.**
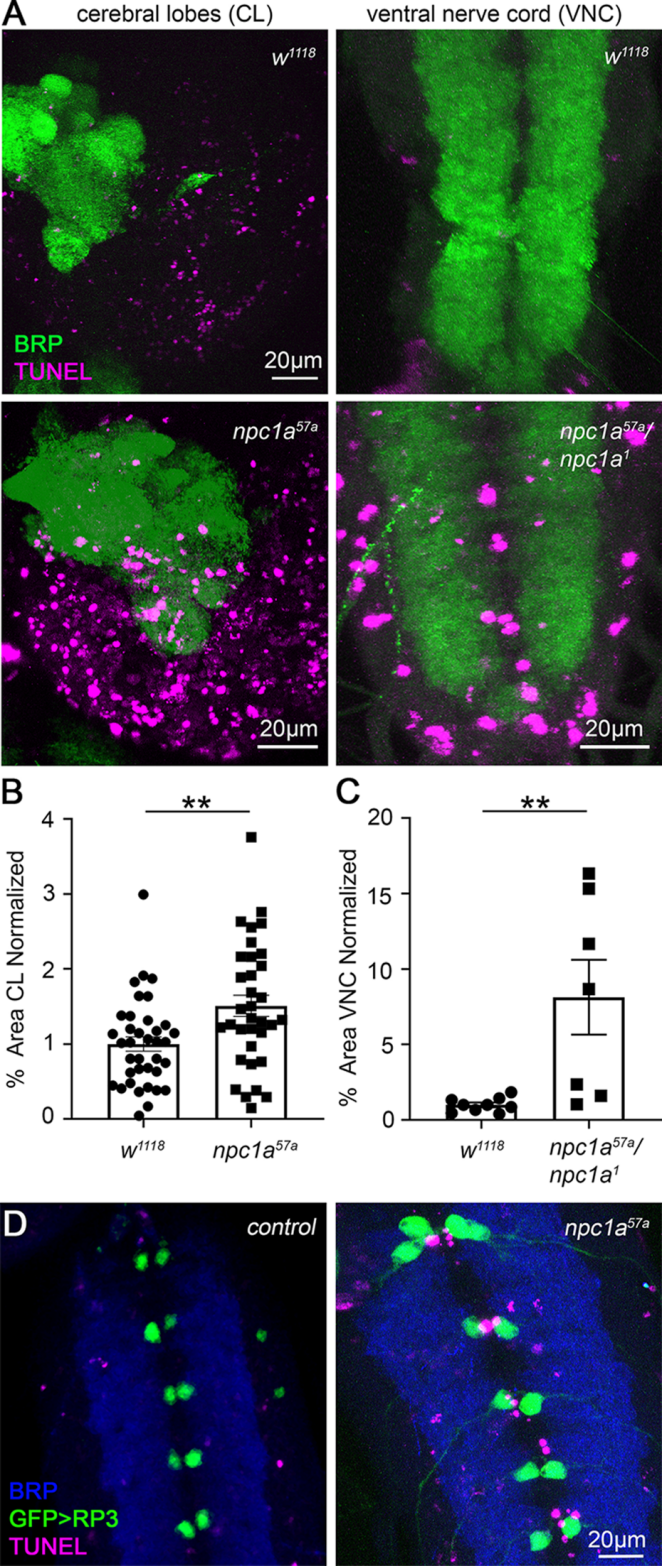
***Npc1a* nulls exhibit central nervous system neurodegeneration, but not in motor neurons.** (A) Representative wandering third-instar larval brain (left) and ventral nerve cord (VNC; right) with terminal deoxynucleotidyl transferase biotin-dUTP nick end labeling (TUNEL, magenta) and synaptic anti-Brp (green) co-labeling in the genetic background control (*w^1118^*, top row) and *Npc1a* null mutants (*Npc1a^57a^* bottom left; *Npc1a^57a^/Npc1a^1^*, bottom right). (B) Quantification of percentage TUNEL area in the cerebral lobes (CL) normalized to *w^1118^* control (*n*=37) for *Npc1a^57a^* mutants (*n*=34) shows a significant increase by Mann–Whitney test (**P=0.0039). (C) Quantification of TUNEL area in the VNC normalized to control (*n*=9) for *Npc1a^57a^/Npc1a^1^* mutants (*n*=7) shows a significant increase by Mann–Whitney test (**P=0.0018). (D) High-magnification VNC images labeled for TUNEL (magenta), Brp (blue) and *CCAP*-Gal4*>UAS-GFP* to mark RP3 motor neurons (green) in control (*CCAP*-Gal4*>*UAS-GFP, left) and mutant (*Npc1a^57a^; CCAP*-Gal4*>*UAS-GFP, right).

**Fig. 7.**
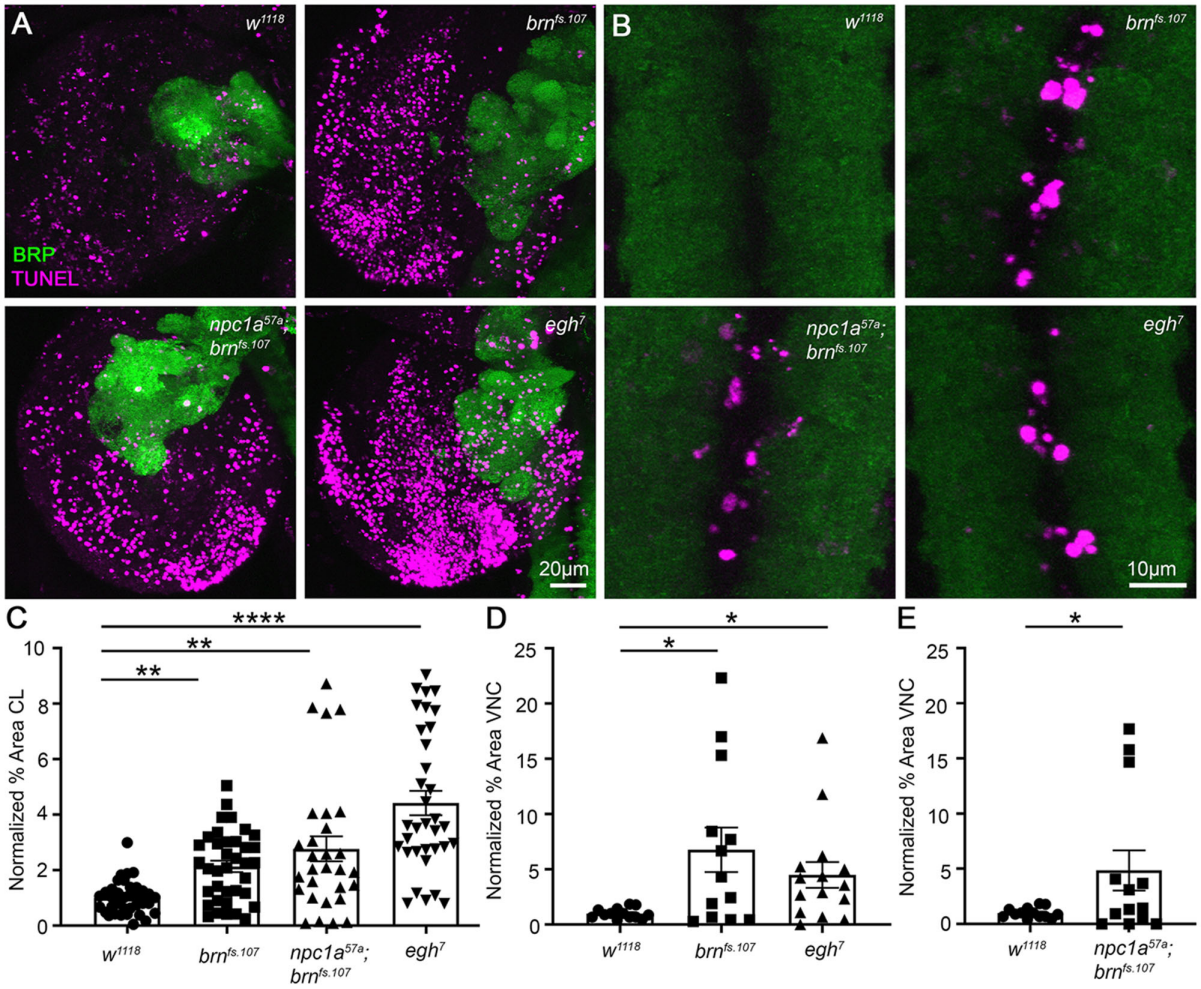
**Glycosphingolipid pathway mutants have increased neurodegeneration.** (A) Representative brain CL co-labeling for TUNEL (magenta) and Brp (green) in genetic control (*w^1118^*), *egh^7^*, *brn^fs.107^* and *Npc1a^57a^; brn^fs.107^* double mutants. (B) VNC co-labeling for TUNEL (magenta) and BRP (green) in the same genotypes. (C) Quantification of percentage TUNEL area in the brain CL normalized to control (*n*=37) for *brn^fs.107^* (*n=37*), *Npc1a^57a^; brn^fs.107^* (*n*=29) and *egh^7^* (*n*=34) shows statistical increases for *brn^fs.107^* (**P=0.0029), *Npc1a^57a^; brn^fs.107^* (**P=0.0013) and *egh^7^* (****P<0.0001) based on a Kruskal–Wallis test with multiple comparisons. (D) Quantification of TUNEL area in the VNC normalized to control (*n*=13) for *brn^fs.107^* (*n*=13) and *egh^7^* (*n*=15) mutants shows significant increases in *brn^fs.107^* (*P=0.035) and *egh^7^* (*P=0.036) by Kruskal–Wallis test with multiple comparisons. (E) Quantification in *Npc1a^57a^; brn^fs.107^* double mutants (*n*=13) normalized to controls (*n*=13) shows a significant increase (*P=0.045) by unpaired two-tailed Student's *t*-test.

To test whether GSL-mediated neurotransmission excitotoxicity is linked to neurodegeneration in our NPC disease model, we next used TUNEL labeling to analyze larval neuronal cell death (Vasudevan and Ryoo, 2016; Vita et al., 2021). The wandering third-instar central nervous system components [brain cerebral lobes and ventral nerve cord (VNC)] were co-labeled for the synaptic marker Brp (Kittel et al., 2006) to delineate central neuropils. Characterized cell death occurs normally in the wandering third instar, especially in the brain cerebral lobes (Pinto-Teixeira et al., 2016; Truman and Bate, 1988). We first tested neurodegeneration overall in our NPC disease model by analyzing the TUNEL-positive area in cerebral lobes and VNC. We next tested neurodegeneration specifically in the RP3 motor neurons innervating muscle 6 from the above electrophysiology studies (Broadie et al., 1993), hypothesizing that neurodegeneration due to synaptic hyperexcitability induced excitotoxicity (Peng et al., 2019). Finally, we tested the GSL pathway mutants for neurodegeneration, including both *egh^7^* and *brn^fs.107^* single mutants, and *Npc1a^57a^; brn^fs.107^* double mutants compared to the genetic background control (*w^1118^*). In all four genotypes, we co-labeled for TUNEL and Brp, analyzing TUNEL-positive area in brain cerebral lobes and VNC. Representative images and data quantification are shown in Figs 6 and 7.

Control *w^1118^* animals exhibit considerable normal TUNEL-labeled neuronal death in the brain cerebral lobes, but very limited neurodegeneration in the VNC (Fig. 6A, top row). In the brain, widespread TUNEL labeling occurs, consistent with neuroblast lineage apoptosis whereby approximately half of all neurons die (Kumar et al., 2009; Pinto-Teixeira et al., 2016). In the VNC, little TUNEL labeling is present around the motor neuron cell bodies along the central midline between the two lateral neuropils (Fig. 6A, top row). In contrast, *Npc1a* null mutants exhibit clearly increased TUNEL labeling in the brain and particularly in the VNC (Fig. 6A, bottom row). The percentage TUNEL-positive area was quantified for cerebral lobes and the central VNC between the lateral neuropils defined by Brp immunofluorescence (Fig. 6A). Normalized to the *w^1118^* genetic controls (1.00±0.096; *n*=37), *Npc1a* nulls showed 50% higher TUNEL area in the brain cerebral lobes (1.509±0.143; *n*=34), which is a significant elevation (*P*=0.0039; Mann–Whitney; Fig. 6B). The elevated cerebral lobe cell death in the NPC model did not appear restricted to any particular brain regions. Normalized to *w^1118^* genetic controls (1.00±0.158; *n*=9), *Npc1a* nulls (*Npc1a^57a^/Npc1a^1^*) showed an 8-fold increase in TUNEL area in the central VNC (8.143±2.479; *n*=7), which is also a significant elevation (*n*=9; *P*=0.0018; Mann–Whitney; Fig. 6C). This central VNC neuronal death between the two lateral Brp-positive neuropils (Fig. 6A, bottom right) excludes neuroblast domains and thus represents neurodegeneration of fully differentiated circuitry.

Given the pronounced TUNEL-labeled cell death along the VNC midline in *Npc1a* mutants, we hypothesized the occurrence of motor neuron death, in line with the elevated NMJ transmission in our NPC model (Fig. 1). To test this hypothesis, the RP3 motor neurons innervating muscle 6 were specifically labeled with *CCAP*-Gal4 driven UAS-eGFP (green; Fig. S6A,B). We co-labeled for TUNEL (magenta) and Brp to mark the neuropil (blue) in *Npc1a^57a^* null mutants compared to controls (Fig. 6D). Control animals (*CCAP*-Gal4>UAS-eGFP) again showed essentially no TUNEL-labeled neuronal cell death along the central VNC midline and no indication of RP3 motor neuron death (Fig. 6D, left). In contrast, the NPC disease model null mutants (*Npc1a^57a^; CCAP*-Gal4>UAS-eGFP) showed extensive TUNEL labeling along the VNC midline, albeit with very limited overlap with RP3 motor neurons (Fig. 6D, right). The colocalization between RP3 motor neuron GFP and TUNEL signal was assayed with overlap analyses (Zen program). This quantification indicated a low level of TUNEL-positive RP3 motor neurons in control animals (colocalization ratio, 0.019±0.014; *n*=11), with a trending increase in the NPC disease model (*Npc1a^57a^*, 0.074±0.039; *n*=10), but no significant change in RP3 motor neuron TUNEL label (*P*=0.0728; Mann–Whitney). This trend suggests that RP3 motor neurons are in the earliest stages of neurodegeneration, but the majority of NPC model TUNEL-labeled cell death along the VNC midline clearly did not occur within these motor neurons.

We next tested GSL pathway mutants. Both *egh^7^* and *brn^fs.107^* mutants showed increased TUNEL labeling in cerebral lobes (Fig. 7A) and VNC (Fig. 7B). The *Npc1a^57a^; brn^fs.107^* double mutants showed no further increase in TUNEL labeling (Fig. 7A,B). Quantification in the cerebral lobes normalized to *w^1118^* controls (1.00±0.0963; *n*=37) revealed greater TUNEL-positive area in *brn^fs.107^* mutants (2.135±0.206; *n*=37; *P*=0.0029), *Npc1a^57a^; brn^fs.107^* double mutants (2.768±0.4511; *n*=29; *P*=0.0013) and especially *egh^7^* mutants (4.417±0.439; *n*=34; *P*<0.0001; Fig. 7C). Importantly, TUNEL labeling in *brn^fs.107^* mutants and *Npc1a^57a^; brn^fs.107^* double mutants was not significantly different, suggesting a common pathway. Quantification in the central VNC normalized to controls (1.00±0.1297; *n*=13) likewise revealed elevated TUNEL in *brn^fs.107^* (6.769±2.012; *n*=13; *P*=0.035) and *egh^7^* (4.490±1.165; *n*=15; *P*=0.036; Fig. 7D) mutants. Quantification normalized to controls (1.00±0.1297; *n*=13) revealed similarly elevated TUNEL area in *Npc1a^57a^; brn^fs.107^* mutants (4.854±1.816; *n*=13; *P*=0.0449; Fig. 7E). Feeding miglustat to *Npc1a^57a^*, *brn^fs.107^* and *egh^7^* mutants did not alter TUNEL labeling in cerebral lobes or VNC (Fig. S7A,B). Quantification of the miglustat-fed mutants showed maintained higher levels of neurodegeneration (Fig. S7C,D). These results indicate that manipulating GSL levels causes neurodegeneration, phenocopying the cell death exhibited in the NPC model (*Npc1a* nulls). Overall, our findings link GSLs to the maintenance of normal levels of neurotransmission and neurodegeneration.

## DISCUSSION

This is the first study to test a link between GSL mechanisms and neurotransmission and neurodegeneration defects in the *Drosophila* NPC disease model. Our previous work in this model demonstrated age-dependent cholesterol accumulation and neurodegeneration in the adult brain, and shortened adult lifespan (Phillips et al., 2008). Others have reported cholesterol accumulation in *Npc1a* null first instars (Huang et al., 2005) and even earlier defects in Hedgehog signaling in mutant embryos (Bialistoky et al., 2019). The current study fills an important gap in third instars, demonstrating elevated neurotransmission and neurodegeneration in *Npc1a* nulls. It has been proposed that ganglioside accumulation is a primary driver for NPC phenotypes (Zervas et al., 2001a,b), which is problematic because *Drosophila* lacks complex gangliosides. However, GSLs may be pathogenic (Wheeler et al., 2019; Zervas et al., 2001a,b), with GSL accumulation prior to cholesterol buildup (Lloyd-Evans et al., 2008), and *Drosophila* is a good model to test GSL pathway mechanisms. For synaptic transmission, a GSL link is shown by the *brn* N-acetylglucosaminyltransferase mutant (Wandall et al., 2005) phenocopy of the *Npc1a* defect, miglustat glucosylceramide synthase inhibitor (Patterson et al., 2007) prevention of *Npc1a* and *brn* defects, and the lack of additive defects in *Npc1a; brn* double mutants. A focus on GlcCer derives from the lack of defect in *egh* mannosyltransferase mutants (Wandall et al., 2005), lack of MacCer changes in *Npc1a* mutants (Huang et al., 2018) and GlcCer accumulation. For neurodegeneration, *Npc1a*, *egh*, *brn* and *Npc1a; brn* double mutants all displayed heightened TUNEL-labeled cell death in the nervous system, with synaptic dysfunction preceding neuronal death, suggesting likely GSL mechanisms in this NPC disease model.

We first found that *Npc1a* mutants have elevated glutamatergic synaptic transmission. In the mouse NPC model, a similar increase occurs in the Schaffer collateral/commissural pathway composed of glutamatergic pyramidal neurons projecting into the hippocampal CA1 region (D'Arcangelo et al., 2016). *Drosophila* NMJ electrophysiology allows muscle intracellular recording in individually identified cells (e.g. RP3 motor neuron to muscle 6) (Broadie et al., 1993), in contrast to the mouse hippocampus extracellular recordings for regional changes (Bykhovskaia and Vasin, 2017; D'Arcangelo et al., 2016). Moreover, directly measuring synaptic defects in the motor circuit may be critical to understanding NPC progression, because motor ataxia is a primary NPC symptom (Patterson et al., 2020). Glutamatergic hyperexcitability may also underlie seizures (Wu et al., 2022), another common NPC symptom (Saini et al., 2019). Additionally, hyperexcitability has been linked to intellectual disability in other neurological disorders (Liu et al., 2022) and could account for the intellectual disability in NPC patients (Patterson et al., 2020). The *Npc1a* NMJ synaptic transmission defect builds on our previous work showing defects in extracellular photoreceptor electroretinogram (ERG) recordings (Phillips et al., 2008). ERG traces show a decrease in synaptic amplitude with advanced neurodegeneration, whereas NMJ recordings show neurotransmission elevation prior to neurodegeneration. In addition to these functional changes, *Npc1a* mutants display overgrown synaptic architecture with increased NMJ area, synaptic bouton number and terminal branch number. Although NMJ structure can sometimes account for functional defects (Budnik et al., 1990; Menon et al., 2013), *Npc1a* mutants do not have increased presynaptic active zone number, suggesting that NMJ growth is independent of the strengthened neurotransmission.

We next found that the *brn* mutant phenocopies the elevated synaptic transmission observed in the *Npc1a* mutant. These *brn* mutants accumulate truncated GSLs and lack elongated GSLs (Wandall et al., 2005) and also exhibit defective *Drosophila* NMJ *trans*-synaptic signaling (Huang et al., 2018). Consistently, *brn* mutant punctate MacCer accumulations localize with both presynaptic (HRP) and postsynaptic (DLG) membrane markers at NMJ synaptic boutons, as well as widely in the surrounding extrasynaptic muscle (Huang et al., 2018). GSL changes in many neurodegenerative disorders involve synaptic transmission defects (Garcia-Ruiz et al., 2015). For example, mutants in serine palmitoyl transferase, which converts L-serine and palmitoyl-CoA to 3-oxosphinganine, show striking GSL changes in juvenile amyotrophic lateral sclerosis, a neurodegenerative disease characterized first by synaptic hyperexcitability and then later motor circuit degeneration (Dodge et al., 2015; Johnson et al., 2021; Lone et al., 2022; Menon et al., 2020). This progression appears to be consistent with the *brn* mutant increase in synaptic transmission. In contrast, we found that *egh* mutants exhibit no change in neurotransmission strength. Likewise, these same *egh* mutants exhibit no change in elongated GSLs, showing a slight increase in GlcCer accumulation but no change in MacCer abundance (Wandall et al., 2005). *egh^62d18^* mutants have aberrant NMJ synaptic architecture, but function was not assayed (Huang et al., 2018), and this allele is sadly now lost. The *brn*-specific function controlling NMJ neurotransmission strength is consistent with known synaptic GSL roles regulating both endocytosis (Sharma et al., 2005) and receptor localization mechanisms in lipid rafts (Roh et al., 2014).

Miglustat prevented the elevated glutamatergic neurotransmission in *Npc1a* mutants. Acting as a GlcCer synthase inhibitor (Patterson et al., 2007; Platt et al., 1994), miglustat is the only approved NPC treatment (European Union only), and miglustat is the only drug treatment shown to stabilize the cognitive decline of NPC patients (Patterson et al., 2020). As in the *Drosophila* NPC model, miglustat feeding prevents the synaptic transmission elevation in the mouse NPC model in the glutamatergic Schaffer collateral/commissural pathway (D'Arcangelo et al., 2016), indicating a conserved GSL mechanism limiting glutamatergic neurotransmission in these two disease models. Moreover, miglustat feeding also prevented *brn* synaptic transmission elevation, further establishing the pathogenicity of intermediate GSLs and providing evidence for a linked pathway between *Npc1a* and the GSL biosynthetic pathway. Previous *Drosophila* studies used the 1-phenyl-2-decanoylamino-3-morpholino-1-propanol inhibitor of GlcCer synthase to show NMJ synaptic architecture changes, with fewer and larger synaptic boutons, but no functional analysis has been reported (Huang et al., 2018). Because miglustat may well have off-target effects, for example inhibiting non-lysosomal glucosyl ceramidase (GBA2), which removes the glucose moiety from GlcCer to convert it back to ceramide in the plasma membrane inner leaflet (Wheeler et al., 2019), it could be useful to test whether PDMP also restores *Npc1a* mutant synaptic transmission. This could help confirm that miglustat prevention of the *Npc1a* neurotransmission elevation is due to GlcCer synthase and not to GBA2 inhibition (Wheeler et al., 2019). Regardless, miglustat correction of the *Npc1a* neurotransmission increase indicates the importance of the GSL synthesis pathway in limiting synaptic function in our NPC disease model.

We next found that neuronally driven *Npc1a* and *brn* RNAi knockdown induces the same neurotransmission elevation, suggesting a presynaptic mechanism. Mouse NPC disease models likewise show presynaptic NPC requirements; however, the phenotypes are consistent with impaired neurotransmission, which may occur in a more advanced state of neurodegeneration. Previous studies report a decreased total synaptic vesicle pool, impaired vesicle release and larger vesicles in primary hippocampal neurons in the NPC mouse model (Mitroi et al., 2019; Hawes et al., 2010; Karten et al., 2006). Importantly, the same study that documented impaired synaptic vesicle release confirmed that the glutamatergic Shaffer collaterals to CA1 hippocampal neurons have an excitatory neurotransmission phenotype (Mitroi et al., 2019), so it is possible that the increase in neurotransmission is maintained by the increase in synaptic vesicle size (Mitroi et al., 2019; Karten et al., 2006), or possibly by increases in presynaptic evoked calcium (Lloyd-Evans et al., 2008). It is also important to note that several postsynaptic glutamatergic receptor defects have been reported in the NPC mouse and rat models. Previous studies have suggested impairment in kainate receptors and an imbalance in AMPA receptors (D'Arcangelo et al., 2011), as well as impaired delivery of AMPA receptors during LTP in the hippocampus in the NPC mouse model (Mitroi et al., 2019). More research is needed on presynaptic and postsynaptic NPC1 roles, especially at early time points in disease model progression. Importantly, neuron-targeted *brn* RNAi phenocopies the *Npc1a* neurotransmission increase, showing that the GSL requirement is also presynaptic and supporting the conclusion that *Npc1a* and *brn* function within the same pathway to increase synaptic transmission.

We next found that *Npc1a; brn* double mutants have elevated synaptic transmission of the same magnitude as the individual *brn* and *Npc1a* single mutants, further indicating that *brn* and *Npc1a* likely function within the same pathway to increase presynaptic neurotransmission. Such double mutant genetic assays are a powerful approach to test whether two genes operate in the same pathway (no additional change) or in different, parallel pathways (additive phenotypes) (Haber et al., 2013; Martínez-Laborda et al., 1996). Importantly, our results indicate that *brn* is not sufficient to alter the elevated synaptic transmission in *Npc1a* nulls, and thus that accumulation of either MacCer or GlcCer may be responsible for the *Npc1a* neurotransmission phenotype. Similarly, although miglustat restoration of neurotransmission in CA1 hippocampal neurons of the mouse NPC model has also shown that the GSL synthesis pathway is important in neurotransmission (D'Arcangelo et al., 2016), the synaptic defect has not been linked to accumulation of a specific GSL species. In NPC clinical treatments, miglustat also acts via an uncharacterized GSL correction (Platt et al., 1994; Zervas et al., 2001a,b). In order to determine whether MacCer or GlcCer causes synaptic dysfunction in our NPC model, we next tested the *Npc1a; egh* double mutant, surprisingly finding no change in the double mutants compared to the *Npc1a* single mutants. Given that the loss of *egh* function is insufficient to restore *Npc1a* synaptic transmission, we cannot make a strong conclusion about the role of GlcCer accumulation in synaptic transmission. We therefore turned to the analysis of synaptic MacCer, which we can image directly, to determine whether MacCer is accumulated as the pathogenic GSL driving the elevated synaptic transmission.

The *Drosophila* NPC disease model shows no increase in synaptic MacCer levels. Our work confirms previous studies that indicate that *brn* mutants, in contrast, display elevated MacCer at the NMJ (Huang et al., 2018). This is surprising given that *Npc1a* and *brn* operate in the same pathway to increase synaptic transmission, and we had hypothesized a parallel increase in synaptic MacCer levels. Human NPC patients and the feline NPC model both show elevated LacCer, a mammalian dihexosylsceramide counterpart to MacCer (Stein et al., 2012; Vanier, 1983; Wandall et al., 2005). However, synaptic MacCer levels do not correlate with elevated synaptic transmission in the *Drosophila* NPC model. The *Npc1a; brn* double mutants do show a dramatic synaptic MacCer elevation, in and around the synapse, but this does not explain the elevated neurotransmission, despite likely MacCer incorporation into important synaptic lipid rafts (Yu et al., 2021). We therefore hypothesize that GlcCer levels are responsible for the *Npc1a* synaptic defect. We cannot visualize synaptic GlcCer, but we did find that GlcCer levels in whole animals are increased based on LC-MS. This GlcCer elevation is conserved across human patients and mouse disease models. NPC patient liver and spleen have a ∼15- to 35-fold GlcCer increase, a greater elevation than for cholesterol, dihexosylceramides and gangliosides (Vanier, 1983). GlcCer levels are also increased in mouse NPC model brain tissue (Fan et al., 2013). GlcCer is implicated in increased calcium response to glutamatergic signaling (Korkotian et al., 1999) and also in heightened agonist-stimulated calcium release occurring via ryanodine receptors (Lloyd-Evans et al., 2003). We therefore suggest that elevated GlcCer levels cause the increased neurotransmission in our NPC disease model.

We next found that *Npc1a* larvae exhibit elevated neurodegeneration. Heightened glutamate neurotransmission drives excitotoxicity (Dong et al., 2009; Peng et al., 2019), which we hypothesized is the causal link. In human NPC patients and cat/mouse models, the disease state is characterized by Purkinje cerebellar neuron cell death (Cougnoux et al., 2016; Elrick et al., 2009; Vite et al., 2015), which is phenocopied by *Drosophila* adult neurodegenerative vacuolization (Phillips et al., 2008). In the larval brain, *Npc1a* mutants show increased cell death in brain cerebral lobes and VNC. To test links between heightened neurotransmission and VNC neurodegeneration, we assayed TUNEL-labeled cell death within the individually identified RP3 motor neurons used in our electrophysiology experiments (Broadie et al., 1993). Although there is a trend towards increased TUNEL immunoreactivity in RP3 neurons, they remain viable with elevated neurotransmission, indicating that synaptic dysfunction precedes neurodegeneration in this circuit. Our previous adult retina work shows that *Npc1a* mutants have decreased synaptic transmission alongside retinal degeneration (Phillips et al., 2008), with impaired synaptic function likely reflecting advanced neurodegeneration. In the mouse NPC model, no electrophysiological defects are reported prior to Purkinje cell neurodegeneration (Elrick et al., 2009), suggesting an apparent difference between inhibitory and excitatory glutamatergic neurons. Importantly, glial death has been reported in the mouse NPC model (German et al., 2002); however, we found no TUNEL-positive glial cells surrounding RP3 neurons, suggesting that the dying cells must instead be interneurons. Previous work suggests that there are decreases in cerebellar interneurons in the *Npc1^−/−^* mouse model (Cougnoux et al., 2020), so our future work will focus on studying interneuron loss mechanisms.

We finally found that *egh* and *brn* mutants have heightened neurodegeneration, just like *Npc1a* mutants, in both cerebral lobes and central VNC containing the motor circuitry. In Gaucher's disease, GlcCer accumulation has been linked to progressive spatial ataxia (Malekkou et al., 2018). In Parkinson's disease, dopaminergic neurodegeneration is also linked to GlcCer dysregulation (Belarbi et al., 2020). Likewise, the motor neuron disorder amyotrophic lateral sclerosis is associated with GlcCer accumulation (Johnson et al., 2021). Therefore, elevated *egh* and *brn* TUNEL-labeled neuronal death may reflect a common cause for numerous neurodegenerative conditions affecting multiple neuronal classes. The *Npc1a; brn* double mutants also exhibit comparable TUNEL-labeled neuronal death in cerebral lobes and VNC. Because *brn* removal does not rescue the *Drosophila* NPC model neurodegeneration, we hypothesize that earlier GSL accumulation (i.e. GlcCer) is causative of neuronal death. However, neurodegeneration in both *Npc1a* and GSL mutants is not prevented by miglustat feeding. A previous study reported that miglustat prolongs survival of Purkinje neurons in the feline NPC model (Stein et al., 2012). Other work has documented a decrease in Purkinje cell loss in human NPC patients with miglustat treatment (Pineda et al., 2018). These differences could indicate that miglustat is an insufficiently potent GlcT1 inhibitor to modulate cell death phenotypes. Alternatively, miglustat dosage/delivery in *Drosophila* may not be comparable to that in humans or cats. Because feeding miglustat to GSL mutants also does not affect neuronal death, phenocopying the NPC model, the drug appears ineffective in modulating GSL-based neurodegeneration. Nevertheless, we conclude that GSL misregulation is clearly linked to neurotransmission upregulation and subsequent neurodegeneration in the *Drosophila* NPC disease model.

## MATERIALS AND METHODS

### *Drosophila* genetics

All mutant and transgenic lines were obtained from the Bloomington *Drosophila* Stock Center (BDSC). All stocks were grown on standard corn meal/molasses food at 25°C. The genetic background control was *w^1118^*. Null *Npc1a* alleles (*Npc1a^1^* and *Npc1a^57a^*) were used in both homozygous and *trans*-heterozygous combinations (Fluegel et al., 2006; Huang et al., 2005). Controls and mutants were fed 0.14 mg/g^−1^ 7-dehydrocholesterol to circumvent the *Npc1a* null mutant lethality (Huang et al., 2005; Phillips et al., 2008). The viable *egh^7^* mutant is a point mutation at amino acid 308 converting methionine to lysine (M308K) (Wandall et al., 2005). The viable *brn^fs.107^* mutant is an intragenic deficiency (Goode et al., 1992). All the double mutants were made using standard genetic crosses. The Gal4 transgenic lines were ring gland driver *2-286*-Gal4 (Phillips et al., 2008), pan-neuronal driver *elav*-Gal4 (BDSC, 8760) and RP3 motor neuron driver *CCAP-*Gal4 (BDSC, 39292). The UAS responder lines were UAS-*npc::YFP* (BDSC, 41762), UAS-*eGFP* (BDSC, 5430), UAS-*Npc1a* RNAi (BDSC, 37504) and UAS-*brn* RNAi (BDSC, 55386). Transgenes were introduced into mutants using standard recombination techniques.

### TEVC electrophysiology

Recording was done as previously described (Bhimreddy et al., 2021; Kopke et al., 2017). Briefly, wandering third instars were dissected along the dorsal midline, and the body wall was glued (3M Vetbond Tissue Adhesive) to a sylgard-coated slide. The internal organs were removed to reveal the neuromusculature, and the peripheral nerves were cut at the VNC exit points. Dissections were done at 18°C in standard saline: 128 mM NaCl, 4 mM MgCl_2_, 1.0 mM CaCl_2_, 70 mM sucrose and 5 mM HEPES (pH 7.2). Dissected preparations were imaged with a Zeiss Axioskop microscope using a 40× water-immersion objective. Ventral longitudinal muscle 6 in abdominal segments 3/4 was impaled by two KCl (3 M)-filled microelectrodes (1 mm outer diameter; World Precision Instruments) with ∼15 M Ω input resistance. The cut motor nerves were sucked into a fire-polished glass suction electrode and stimulated using 0.5 ms suprathreshold voltage stimuli at 0.2 Hz (Grass S88 stimulator) to generate EJC recordings. Nerve stimulation-evoked EJC recordings were filtered at 2 kHz. To quantify EJC amplitude, ten consecutive traces were averaged to produce a mean peak value. Data were acquired with Clampex 9.0 and analyzed with Clampfit 9. Miglustat (MedChem Express) in dimethyl sulfoxide (Thermo Fisher Scientific) was fed at concentrations of 0.1-10 ng/ml (Mitroi et al., 2019).

### Confocal immunocytochemistry

Antibody labeling was done as previously described (Bhimreddy et al., 2021; Kopke et al., 2017). Briefly, wandering third instars were dissected as above, then fixed with 4% paraformaldehyde (PFA). For mouse anti-Brp [1:200; Developmental Studies Hybridoma Bank (DSHB), nc82], rabbit anti-repo (1:1000; gift from Dr Benjamin Altenhein, University of Cologne, Germany; Sieglitz et al., 2013) and mouse anti-DLG (1:200; DSHB, 4F3), samples were fixed in PFA for 10 min. For mouse anti-MacCer (Wandall et al., 2005) and Cy-3 conjugated goat anti-HRP (1:200; The Jackson Laboratory, 123-605-021), samples were fixed in PFA for 60 min (Huang et al., 2018). Samples were washed in phosphate-buffered saline (PBS) with 0.2% Triton X-100 (PBT) and 0.5% bovine serum albumin (BSA), 3× for 10 min each. Samples were incubated with primary antibodies overnight at 4°C, followed by PBT washes 3× for 10 min each. Secondary antibodies used were as follows: Alexa Fluor 488 goat anti-mouse (1:250; DSHB, A11001), Alexa Fluor 488 goat anti-rabbit (1:1000; DSHB, A11008), Alexa Fluor 488 donkey anti-rabbit (1:250; DSHB, A21206), Alexa Fluor 555 donkey anti-mouse (1:250; DSHB, A31570), and Alexa Fluor 633 goat anti-mouse (1:250; DSHB, A21052). Samples were incubated with secondary antibodies for 2 h at room temperature. Samples were washed in PBT 3× for 10 min each, and then mounted in Fluoromount G (Electron Microscopy Sciences). Confocal imaging was done as previously described (Bhimreddy et al., 2021; Kopke et al., 2017). Briefly, all imaging was done on a Zeiss LSM 510 META laser-scanning confocal microscope, with images projected in Zen (Zeiss) and analyzed using ImageJ (NIH open source). NMJ measurements were made with the HRP signal to delineate *z*-stack areas of maximum projection using the threshold and wand-tracing tools in ImageJ. All imaging setting were kept constant for comparisons within every set of experiments.

### Lipid mass spectrometry

Optima LC-MS reagents including methanol (CH_3_OH), acetonitrile (CH_3_CN), water (H_2_O), formic acid, isopropyl alcohol, chloroform (CHCl_3_), ammonium acetate, ammonium bicarbonate, ammonium formate (Thermo Fisher Scientific) and tert-butyl methyl ether (MTBE; Sigma Aldrich) were used for LC-MS analyses. Global untargeted lipidomics was performed in the Center for Innovative Technology at Vanderbilt University. Five samples each of *w^1118^* and *Npc1a^57a^* genotypes were prepared by homogenizing whole third-instar larvae weighing 30-50 mg, rinsing with 50 mM ammonium formate and flash freezing in liquid nitrogen with storage at −80°C. Samples were thawed on ice with 0.5 ml of cold CH_3_OH:CH_3_CN:H_2_O (1:1:2, v:v:v) with 50 mM ammonium bicarbonate (lysis buffer), and sonicated by probe tip for 10 pulses at 50% power, followed by vortex mixing for 10 s. Normalized volumes based on the initial dry larvae weights were aliquoted and diluted to 200 µl with lysis buffer followed by an addition of 800 µl CH_3_OH. Samples were vortexed for 30 s and incubated at −80°C overnight for protein precipitation. Samples were then centrifuged at 4°C and 21,000 ***g*** for 15 min, and the supernatant was dried using a cold vacuum centrifuge. Dried samples were reconstituted in 100 µl H_2_O followed by 100 µl CH_3_OH and 10 µl SPLASH® LIPIDOMIX® Mass Spec Standard (Avanti Polar Lipids), and incubated for 10 min at room temperature. Next, liquid–liquid extraction was performed by adding 800 µl MTBE with 30 s of vortex mixing, followed by 10 min incubation on ice and centrifugation at 4°C and 21,000 ***g*** for 15 min. The hydrophobic supernatant (800 µl) was isolated and dried using a cold vacuum centrifuge, and extracts were stored at 4°C. Dried lipid fractions were reconstituted in 100 µl CH_3_OH:CHCl_3_ (9:1, v:v) with exogenous sphingolipid standards (*n*=2) to assess instrument variability. Equal volumes (25 µl) of each sample were combined to create a pooled quality control (QC). Samples were analyzed using an Agilent 1290 LC system interfaced with an Agilent 6560 Ion Mobility Quadrupole Time-of-Flight (Song et al., 2023). Briefly, injection volume was 5 µl with needle wash enabled in the autosampler, which was held at 4°C. A Hypersil Gold column (1.9 µM, 2.1 mm×100 mm, Thermo Fisher Scientific) at 40°C was used for chromatographic separation. The liquid chromatography gradient was 30 min with a flow rate of 0.25 ml/min. Mobile phase A was 10 mM ammonium acetate and 0.1 formic acid additives in H_2_O. Mobile phase B was 10 mM ammonium acetate and 0.1 formic acid additives in CH_3_CN:IPA:H_2_O (60:36:4, v:v:v). Electrospray ionization (Dual AJS, Agilent) was operated in positive polarity with sheath gas flow at 11.8 l/min, sheath gas temperature of 300°C, capillary at 3500 V, nozzle at 2000 V, octupole radio frequency at 750 peak-to-peak voltage (V_pp_), drying gas temperature of 280°C, drying gas flow of 5 l/min and nebulizer at 10 psi. Agilent MassHunter Acquisition software (B.09) was used to operate the instrumentation and to acquire liquid chromatography–ion mobility–tandem mass spectrometry (LC-IM-MS/MS) data in the 75-1700 m*/z* range. A QC sample was injected seven times before the first sample to condition the liquid chromatography column and then injected after every five samples to assess instrument reliability through principal component analysis. Samples were injected randomly with 10% of samples reinjected for quality control purposes. Calibration solution (Agilent Tuning Mixture) was measured at the beginning and end of analysis for mass error quality assurance. Solvent blanks were used to monitor for spectral contaminants and to generate a tandem mass spectrometry (MS/MS) exclusion list. Fragmentation spectra was acquired for the pooled QC via three iterative, top two, data-dependent MS/MS acquisitions. Progenesis QI software (version 3.0, Nonlinear Dynamics, Newcastle, UK) was used to perform data analysis including retention time alignment, peak picking and deconvolution, abundance normalization and one-factor ANOVA. Lipid annotations were performed with reference to in-house and online databases MS-DIAL (Tsugawa et al., 2015), LipidMatch (Koelmel et al., 2017) and Lipid Annotator (Koelmel et al., 2020) using the classification system previously described (Schrimpe-Rutledge et al., 2016). Both HexCer species described in this study were annotated with level 2 confidence, which are putative annotations supported by precursor mass and MS/MS data.

### TUNEL labeling

TUNEL labeling was done according to manufacturer instructions (Roche, 12156792910) to assay neuron apoptosis as previously described (Vita et al., 2021). Briefly, staged larvae were dissected, fixed and permeabilized as above. Preparations were incubated in a thermocycler in 10% PBT and 100 mM sodium citrate (1:100 mix) at 65°C for 30 min, and then washed in PBT 3× for 10 min each. Preparations were incubated in 45 μl labeling solution at 37°C for 30 min. Then, 5 μl of the labeling enzyme was added, and preparations were then incubated at 37°C for 2 h. Preparations were washed 3× for 10 min each in PBT, mounted in Fluoromount, and imaged as above. For TUNEL imaging, *z*-stacks were collected from brain cerebral lobes, and the central VNC was delineated by Brp labeling. Representative maximum-intensity projection images displayed contain all TUNEL signal from each *z*-slice. For TUNEL/Brp images, maximum-intensity projection files were analyzed unaware of genotype. For quantification, the whole area was selected, excluding the Brp-positive neuropil. Data were binarized using the threshold tool in ImageJ, and the percentage area of TUNEL labeling was measured. To label only the RP3 motor neurons (Broadie, et al., 1993), the *CCAP-*Gal4 driver was used to express UAS-eGFP. Images were analyzed unaware of genotype with Zen software for colocalization of TUNEL and eGFP in every *z*-stack optical section, with colocalization confirmed in orthogonal views.

### Statistical analyses

All statistical analyses were performed using Prism software (GraphPad, version 7.04). All data were subject to D'Agostino-Pearson normality tests. If the data fell within the Gaussian distribution, a ROUT (Q=1%) outlier test was performed. All comparisons between two experimental groups were performed using a parametric unpaired two-tailed Student's *t*-test or a non-parametric Mann–Whitney test. All comparisons between three or more experimental groups were analyzed with a one-way ANOVA or non-parametric Kruskal–Wallis test. All graphs were made using Prism software to show all individual data points normalized to the control. All data in this study were collected and analyzed continuously. All graphs show the mean±s.e.m., with significance indicated as *P<0.05, **P<0.01, ***P<0.001 and *P*>0.05 [not significant (ns)].

## Supplementary Material

10.1242/dmm.050206_sup1 Supplementary information
