# Supplementary material for: Glycosphingolipids are linked to elevated neurotransmission and neurodegeneration in a Drosophila model of Niemann Pick type C
Source: Dis Model Mech. 2023 Oct 12;16(10):dmm050206. doi: 10.1242/dmm.050206 (PMC10581387; doi:10.1242/dmm.050206)
Supplement: Supplementary information [file dmm-16-050206-s1.pdf]

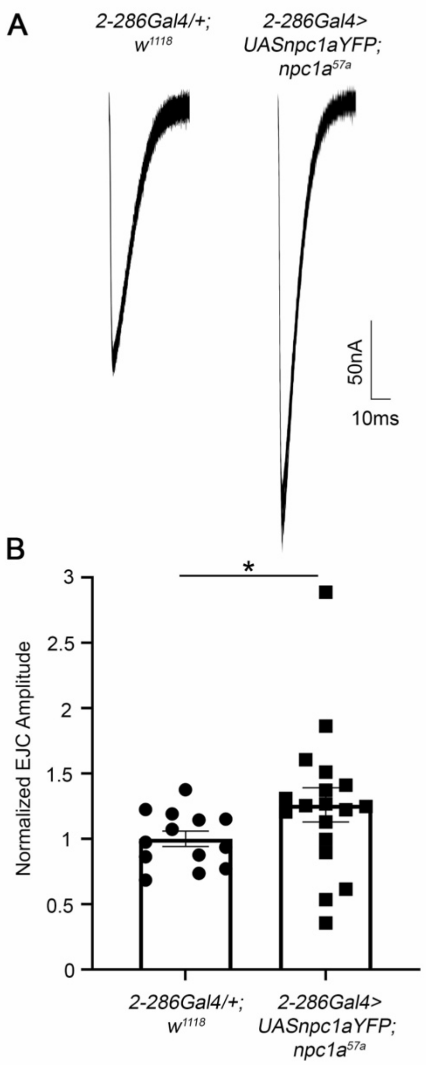

Fig. S1. Ring gland rescued *npc1a* mutants display elevated neurotransmission

A) Representative excitatory junction current (EJC) traces showing ten superimposed two-electrode voltage-clamp (TEVC) recordings from the ring gland Gal4 driver control (*2-286-Gal4*/w^{1118}, left) compared to the homozygous *npc1a* null mutant with a ring gland driven *npc1a* (*npc1a*^{57a}/*npc1a*^{57a}; *2-286-Gal4> UAS-npc1a::YFP*). **B)** Quantified EJC amplitudes normalized to the control (n=13) compared to the ring gland rescued condition (n=18). Mann-Whitney tests show that the ring gland rescued *npc1a* mutant remains significantly elevated (*) compared to control (p=0.0464).

Disease Models & Mechanisms • Supplementary information

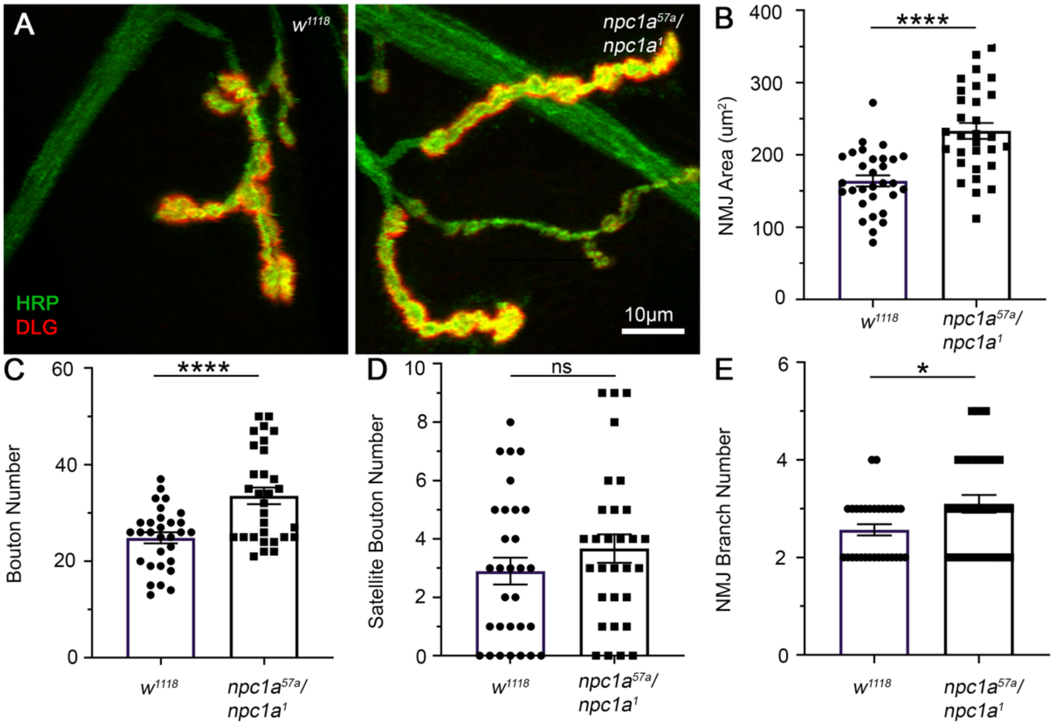

Fig. S2. Null *npc1a* mutants display structural synaptic overgrowth at the NMJ

A) Representative NMJs co-labeled with the synaptic membrane marker anti-horse radish peroxidase (HRP, green) and synaptic scaffold anti-Discs Large (DLG, red) in the genetic background control (w^{1118}, left) and npc1a null ($npc1a^{57a}/npc1a^{1}$, right). **B)** Quantification of HRP-defined NMJ area shows significant increase (****) in *npc1a* mutants compared to controls based on a t-test (n=30 for both; p<0.0001). **C)** Quantification of synaptic bouton number shows significant increase (****) in *npc1a* mutants compared to controls based on a t-test (p<0.0001). **D)** Quantification of satellite bouton number shows no significant (ns) change. **E)** Quantification of NMJ branch number shows a significant increase (*) in *npc1a* mutants compared to controls based on a t-test (p=0.0159).

Disease Models & Mechanisms • Supplementary information

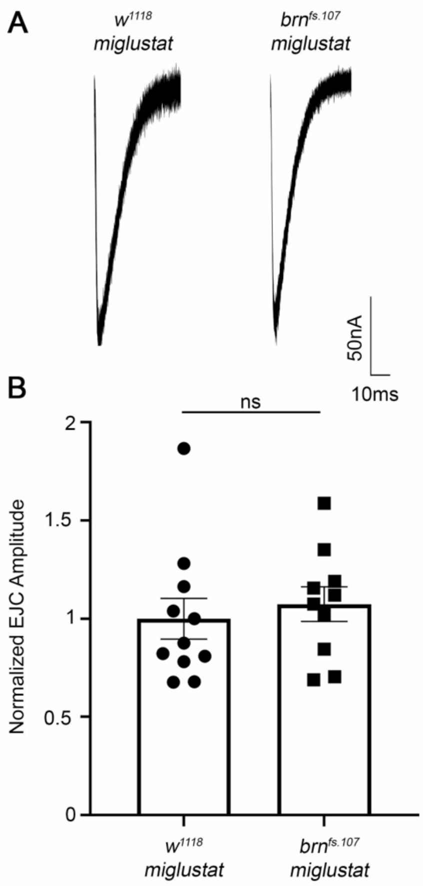

Fig. S3. Miglustat restores *brn* mutant neurotransmission strength to control levels

A) Representative EJC traces from wandering third instar NMJ of the genetic background control (w^{1118}, left) and *brainiac* mutants ($brn^{fs.107}$, right) fed from hatching with miglustat (10 ng/ml) to inhibit glucosylceramide synthase. **B)** Quantification of EJC amplitudes normalized to the w^{1118} control on miglustat (n=11) compared to $brn^{fs.107}$ on miglustat (n=10) shows no significant (ns) difference (p=0.3494).

Disease Models & Mechanisms • Supplementary information

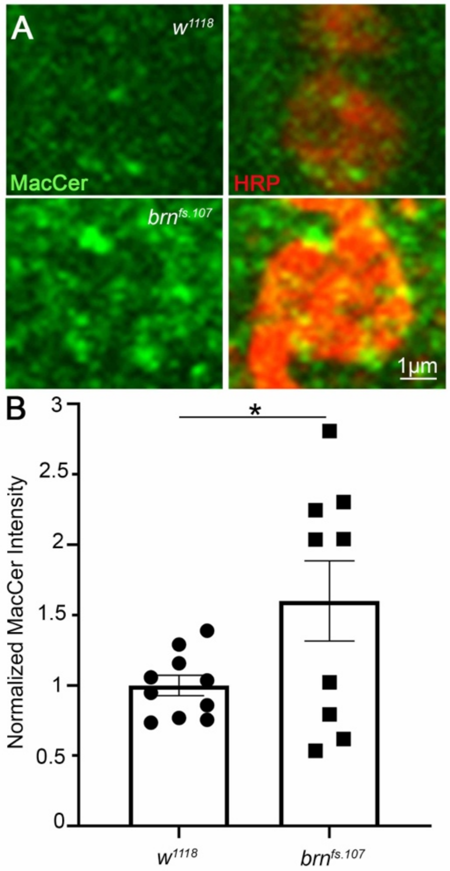

Fig. S4. *brn* mutants display elevated synaptic MacCer accumulation at the NMJ

A) Representative confocal images of anti-mannosylglucosylceramide (MacCer, green) co-labeled with the synaptic membrane marker anti-horse radish peroxidase (HRP, red) at the wandering third instar neuromuscular junction (NMJ) in the genetic background control (w^{1118}, top) and $brn^{fs.107}$ mutant (bottom). MacCer labeling alone (green) is shown on the left, and with the HRP synaptic marker (red) on the right. **B)** Quantification of MacCer fluorescent intensity normalized to the w^{1118} control (n=10) shows that $brn^{fs.107}$ mutants (n=9) are significantly elevated (*) compared to control (p=0.0465).

Disease Models & Mechanisms • Supplementary information

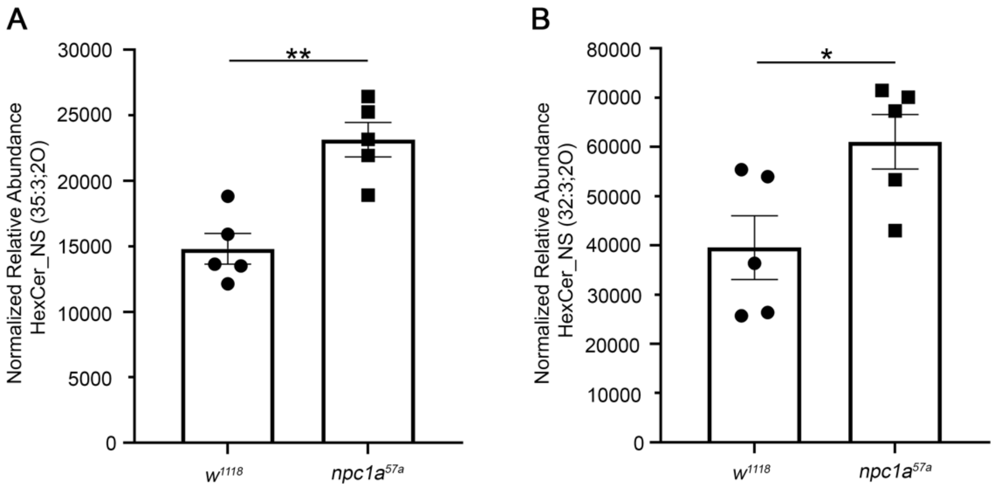

Fig. S5. Mass spectrometry shows *npc1a* mutants display elevated HexCer levels

Lipid mass spectrometry paired with liquid chromatography in positive ion mode used to assess lipid levels of wandering third instar larva in the w^{1118} control and $npc1a^{57a}$ mutant. **A)** Normalized levels of HexCer_NS (35:3;2O) in wandering third instars are significantly increased (**) in $npc1a^{57a}$ mutants (n=5) compared to w^{1118} genetic background controls (n=5) based on a one-way ANOVA (p=0.00157). **B)** Normalized levels of HexCer_NS (32:3;2O) in in wandering third instars are also significantly increased (*) in the $npc1a^{57a}$ mutants (n=5) compared to w^{1118} controls (n=5) based on a one-way ANOVA (p=0.0407).

Disease Models & Mechanisms • Supplementary information

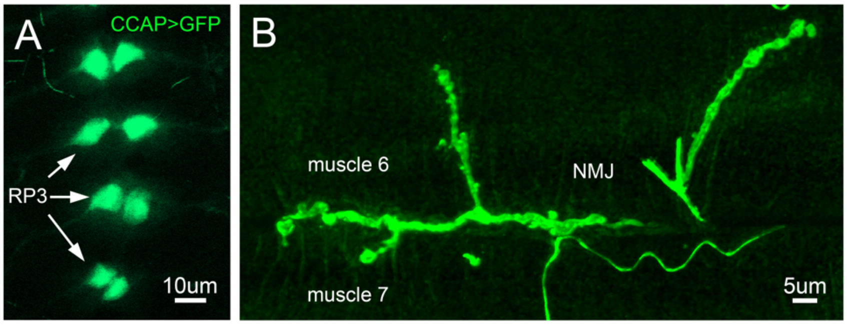

Fig. S6. *Ccap-Gal4* driver is specific to RP3 motor neurons innervating muscles 6/7

A) High magnification image of the ventral nerve cord (VNC) midline in a wandering third instar with *ccap*-Gal4 driving *UAS-GFP* (green). The pair of raw prawn 3 (RP3) motor neurons in each of four segments are highlighted with white arrows. **B)** Representative neuromuscular junction (NMJ) image of RP3 innervating the ventral longitudinal muscles 6 and 7 with *ccap*-Gal4 driving *UAS-GFP* (green).

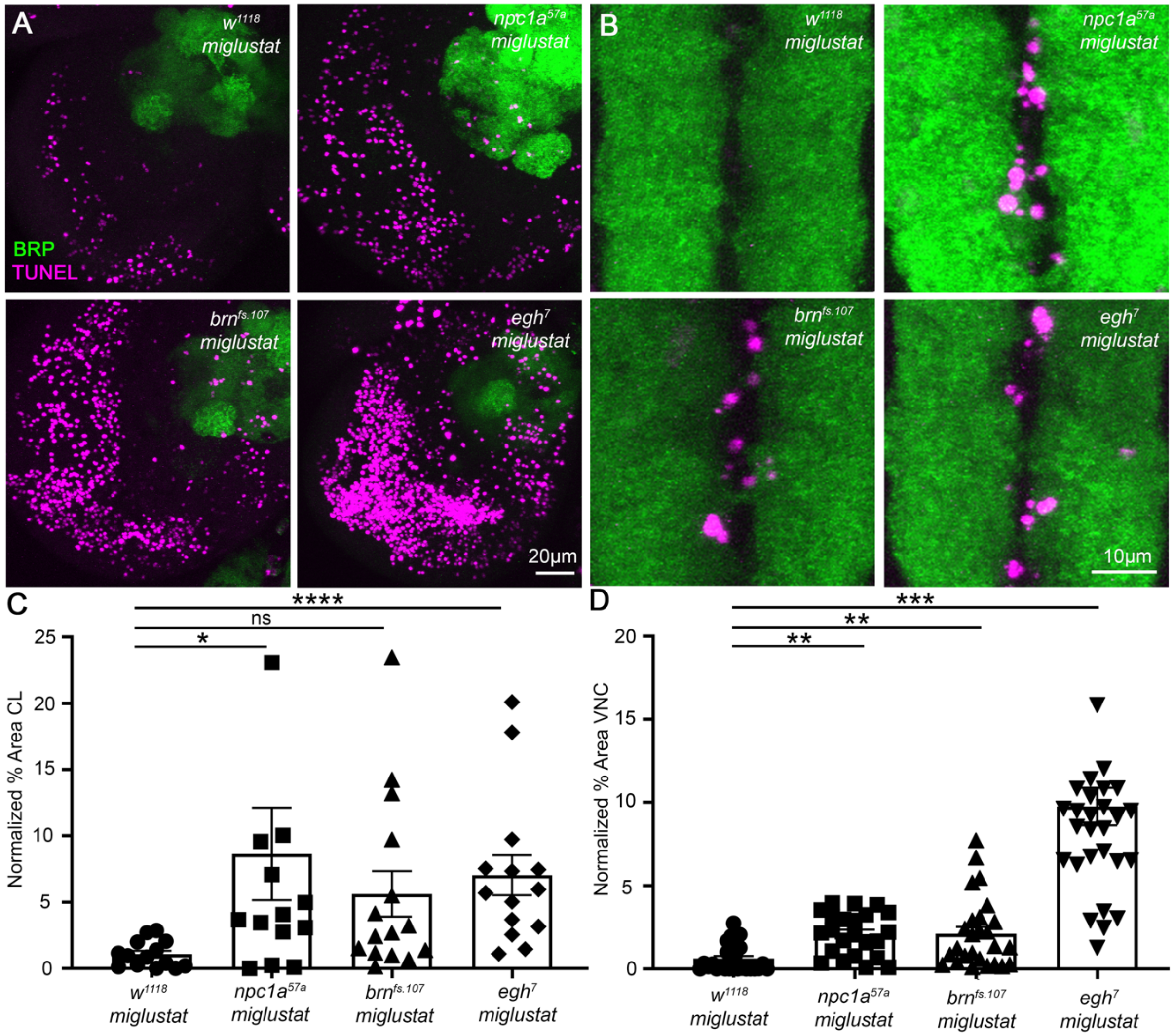

Fig. S7. Miglustat feeding does not prevent neuron death in *npc1a* or GSL mutants

A) Representative brain cerebral lobe (CL) co-labeling for TUNEL (red) and BRP (green) in miglustat-fed genetic control (w^{1118}), $npc1a^{57}$, $brn^{fs.107}$, and egh^{7}. **B)** Ventral nerve cord (VNC) labeling in the same conditions. **C)** Quantification of CL percent TUNEL area normalized to control (n=26) for $npc1a^{57a}$ (n=23), $brn^{fs.107}$ *(n=26), and* egh^{7} (n=30) shows significant increases for $npc1a^{57a}$ (*; p=0.0251) and egh^{7} (****; p<0.0001), but not $brn^{fs.107}$ (ns; p=0.2734) from Kruskal Wallace test with multiple comparisons. **D)** Quantification of VNC percent TUNEL area normalized to control (n=14) for $npc1a^{57a}$ (n=14), $brn^{fs.107}$ (n=15) and egh^{7} (n=14) shows significant increases for $npc1a^{57a}$ (**; p=0.0036), $brn^{fs.107}$ (**; p=0.0046) and egh^{7} (***; p=0.0001) from Kruskal Wallace test with multiple comparisons.
